# Giant-cell arteritis related strokes: scoping review of mechanisms and rethinking treatment strategy?

**DOI:** 10.3389/fneur.2023.1305093

**Published:** 2023-12-07

**Authors:** Mickael Bonnan, Stephane Debeugny

**Affiliations:** ^1^Service de Neurologie, Hôpital Delafontaine, Saint-Denis, France; ^2^Département d'Information Médicale, Centre Hospitalier de Pau, Pau, France

**Keywords:** giant cell (temporal) arteritis, stroke, stroke/physiopathology, embolic stroke, thrombotic stroke

## Abstract

Stroke is a rare and severe complication of giant cell arteritis (GCA). Although early diagnosis and treatment initiation are essential, the mechanism of stroke is often related to vasculitis complicated by arterial stenosis and occlusion. Its recurrence is often attributed to early steroid resistance or late GCA relapse, so immunosuppressive treatment is often reinforced. However, many questions concerning the mechanisms of stroke remain elusive, and no review to date has examined the whole data set concerning GCA-related stroke. We therefore undertook this scoping review. GCA-related stroke does not necessarily display general signs and inflammatory parameters are sometimes normal, so clinicians should observe caution. Ischemic lesions often show patterns predating watershed areas and are associated with stenosis or thrombosis of the respective arteries, which are often bilateral. Lesions predominate in the siphon in the internal carotid arteries, whereas all the vertebral arteries may be involved with a predominance in the V3-V4 segments. Ultrasonography of the cervical arteries may reveal edema of the intima (halo sign), which is highly sensitive and specific of GCA, and precedes stenosis. The brain arteries are spared although very proximal arteritis may rarely occur, if the patient has microstructural anatomical variants. Temporal artery biopsy reveals the combination of mechanisms leading to slit-like stenosis, which involves granulomatous inflammation and intimal hyperplasia. The lumen is sometimes occluded by thrombi (<15%), suggesting that embolic lesions may also occur, although imaging studies have not provided strong evidence for this. Moreover, persistence of intimal hyperplasia might explain persisting arterial stenosis, which may account for delayed stroke occurring in watershed areas. Other possible mechanisms of stroke are also discussed. Overall, GCA-related stroke mainly involves hemodynamic mechanisms. Besides early diagnosis and treatment initiation, future studies could seek to establish specific preventive or curative treatments using angioplasty or targeting intimal proliferation.

## 1 Introduction

Giant cell arteritis (GCA), formerly called *granulomatous arteritis*, is a form of vasculitis involving the large and medium-sized arteries branching to the aorta. GCA is the most frequent form in Western countries. Its incidence increases sharply after the fifth decade to 10.9 per 100.000 inhabitants per year (1), and the lifetime risk prevalence is about 1% in women and 0.5% in men (2). Clinical severity is driven by ischemic complications occurring in the cranial arteries due to active recurrence, sequalae and increased risk of death [up to 28% compared with non-stroke GCA patients (3–7)].

Ophthalmic ischemia is the most common complication. In historical series, bilateral blindness occurred in up to 38% of GCA patients (8–10), whereas its incidence dramatically decreased in the era of steroid treatment down to <3% of monocular blindness (11–13). On the other hand, the incidence of GCA-related stroke at onset has always been low in the range of 2–6% (4, 14–17), and mainly involves the vertebrobasilar territories (4, 15).

A metanalysis confirmed a slightly higher risk of stroke (odds ratio ~1.4) in GCA patients than in age-matched controls (18), and the vasculitis process itself is considered to be the main cause of stroke (15). However, comorbid conditions are frequently found in this age group, although their prevalence is roughly similar with age-matched stroke-free GCA (4, 19, 20), even though this point remains controversial (15, 16). The mechanisms involved in GCA-related stroke are sometimes difficult to ascertain and should not be confused with events occurring frequently in aged patients (3, 21, 22).

Series and reviews of intracranial GCA focused on the main clinical and predictive features of intracranial lesions (4, 5, 17, 23–30), but none of them examined stroke mechanisms in detail, and most data about GCA-related stroke derive from unrelated or heterogeneous case reports that were never systematically examined. Pathological examination of arteries involved in GCA reveals an extended range of transmural inflammation, with granuloma reducing the inner caliber of the artery. The endothelium is mostly spared by inflammation but clots are sometimes observed inside the reduced lumen. Cervical arteritis may extend to the internal carotid and vertebral terminations, the basilar artery and rarely to the very proximal M1 branches, whereas the distal branches are always spared. However, it remains unclear how GCA is related with brain symptoms, resistance to steroid, and stroke relapse. This paper examines questions concerning GCA-related stroke that have received little attention until now:

1) Is there a pattern of stroke associated with GCA? Since extracranial involvement is associated with stenosis/occlusion, stroke involving watershed areas and hemodynamic mechanisms should be over-represented.
2) What is the most distal boundary of GCA-related arterial lesions, and what is the link with internal elastic limitans or the vasa vasorum?
3) Response to steroid initiation is often described as paradoxical since the frequency of stroke relapse may increase within the first days of drug initiation. Delayed stroke suggests resistance to drug treatment, clinical relapse requiring reinforcement of treatment, or unrelated events. Briefly, can the natural history of critical arterial stenosis persisting from GCA relapse be characterized?
4) The diagnosis of cranial involvement in GCA is challenged by the fact that some arteries are spared, and by the lower frequency of general signs or normal inflammatory parameters, especially in ophthalmology. What is the proportion of these 'cold' or misleading cases among patients with GCA-related stroke?
5) What is the kinetics of stenosis, and in turn the kinetics of involution of the arterial inflammation? What is the frequency of persisting stenosis and occlusion?
6) Since a hemodynamical mechanism is the most plausible, can stenting prevent dramatic stenosis and stroke?

We do not deal with the general signs and management of GCA [see Mollan et al. (31)], the mechanisms and clinical management of ophthalmic lesions [see Mollan et al. (32)], nor temporal artery biopsy (TAB) sensitivity. However, data obtained from ophthalmologic studies were used to throw light on the mechanisms of GCA-associated stroke.

## 2 Methods

PubMed request was: (*temporal arteritis* OR *giant-cell arteritis*) AND (*stroke* OR *brain* OR *cerebral* OR *cerebellar* OR *carotid* OR *basilar* OR *vertebral* OR *watershed*). All abstracts of selected articles were read. All articles dealing with the questions raised in the introduction were carefully read and possibly used in the literature review. References of articles were also examined for unnoticed related articles. For statistical methods, see Supplementary material 2.

## 3 Results

### 3.1 Establishing GCA diagnosis in presenting strokes

#### 3.1.1 Definitions

GCA is called *temporal* arteritis, or *cranial* arteritis (as opposed to *extracranial* involvement), which hampers the distinction with brain involvement. Involvement of the cervical non-temporal or intracranial arteries is often not clearly described among admitted GCA extensions (solitary cranial or extracranial, mixed, see Supplementary Table S1). Various terms were used to define this involvement: *intracranial involvement* is based on the location of the ischemic consequence (includes brain stroke but remains unclear about ophthalmic lesions), whereas other authors preferred to use *intra-* or *extra-dural artery lesions* based on the location of putative mechanisms (33). We use the term *cervical arteries* to define arteries abutting to the brain (e.g., carotid or vertebral). Otherwise, strict artery nomenclature was preferred to avoid confusion.

#### 3.1.2 Limitations of ACR criteria applied early

GCA diagnosis is based on the ACR 1990 criteria (34) which were revised in 2022 (35). Cranial ischemic signs were included (jaw/tongue claudication, sudden visual loss) but stroke was excluded (Supplementary Tables 1, 2). Patients presenting at onset with GCA-related stroke before the GCA diagnosis has been made may not meet the criteria due to their lower sensitivity.

In series of patients with ischemic complications (Table 1), new onset localized headache was observed in 74% of patients and temporal artery signs in 56%. The prevalence of abnormal inflammatory parameters may be lower than in non-ischemic GCA patients but the issue is still elusive (often expressed as median compared to non-ischemic patients). When the diagnosis is evoked, TAB gives definitive positive arguments for the diagnosis of GCA. However, the sensitivity of TAB is lower in patients recruited due to ischemic complications in fast-track series (46%−56%) (12, 41). The prevalence of the ACR 1990 criteria cannot be assessed owing to a circularity bias, and no data is available about the 2022 criteria. Moreover, since the sensitivity of fulfilled ACR criteria is high but incomplete (~93%), they are required for clinical research but not for clinical diagnosis (e.g., typical GCA may be diagnosed in a 48-year-old patient). Clinicians faced with stroke should observe caution, although the incidence of GCA-related stroke remains low. The absence of typical GCA signs should not rule out the hypothesis and FDG-PET and/or US of the cranial arteries should be performed owing to their diagnostic accuracy in this setting.

**Table 1:** Prevalence of ACR criteria 1990 in series of ischemic case.

**References**	**Ischemic cases^‡^, *n***	**New onset localized headache, %**	**TA tenderness or decreased arteria pulse, %**	**Positive TAB, %**	**≥3 ACR criteria, %**
Chazal et al. (5)	14	71	n/a	79	^a^
Cid et al. (36)	32	81	94	^a^	n/a
de Boysson et al. (4)	40	73	53	83	^a^
de Mornac et al. (37)	39	49	15	71	^a^
Gonzalez-Gay et al. (38)	69	86	76	n/a	98^b^
Gonzalez-Gay et al. (15)	8	63	75	^a^	100
Nesher et al. (19)	43	67	n/a	81	^a^
Pariente et al. (6)	18	83	33	78	^a^
Pego-Reigosa et al. (39)	30	87	73	^a^	n/a
Sun et al. (40)	29	72	28	n/a	n/a
Zenone and Puget (17)	6	83	0	83	^a^
**Total**	**328**	**74**	**56**	**78**	**—**

^‡^variable criteria of inclusion, see Supplementary material.

^a^criterium for inclusion in study. ^b^not specified in the ischemic group.

n/a, not assessed; TA, temporal artery.

### 3.2 Frequency of stroke and cervical artery lesions

GCA is a rare cause of stroke, accounting for 0.15% to 0.4% of patients admitted for stroke and systematically checked by ultrasonography for “halo sign” (1, 42–44) (Table 2). The incidence of GCA-related stroke over the age of 50 is 0.76 per 100.000 inhabitants per year (1). Stroke is also admitted to be a rare complication of GCA, estimated at below 16%, and congruent for the range of 2–3% of GCA over the series defined in the period extending from GCA onset to the first month following steroid initiation (4, 6, 14–17, 28, 37).

**Table 2:** Red flags for the diagnosis of GCA in stroke.

**Headache or tenderness of temporal artery**
Constitutional symptoms such as fever, malaise, or weight loss
Bilateral occlusions of severe stenosis of the vertebral arteries
Imaging studies showing involvement restricted to extracranial vessels (except carotid bulb) and not the intracranial segments

Modified from Zwicker et al. (45) and Kong et al. (46).

Cervical arteries are very often involved in GCA, either in isolation (37%), or in association with large vessels [42%, in De Mornac et al. (37)], which contrasts with the rarity of observed stroke and suggests that the cervical arteries involved are not so prone to distal complications. However, ischemic complications were possibly underestimated in older series in which stroke was diagnosed on clinical grounds without systematic MRI screening, whereas systematic screening for silent stroke in recent series led to an increased prevalence in non-selected GCA patients [up to 15% in Siemonsen et al. (47)].

A systematic autopsy series found 0.4% of (unsuspected) aortic GCA in the general population (48), suggesting that GCA could be more prevalent than previously thought. However, no data supports the hypothesis of serendipitous cranial GCA underlying stroke.

### 3.3 Lessons from non-brain arteries

We briefly examine mechanism of ischemic manifestations in the eyes and limbs and show that low flow is the main problem whereas embolic events are mostly absent.

#### 3.3.1 Lessons from optic ischemia

Our study did not focus upon ophthalmic signs, but the frequency of optic involvement is higher than stroke and probably shares similar mechanisms. Ophthalmic, post-ciliary and circles of Zinn arteries are often involved in GCA arteritis (9). In angiography, massive delayed choroidal filling is characteristic of GCA, but various patterns of ischemia are possible: choroidal ischemia, arteritic anterior ischemic optic neuropathy (AAION), posterior ischemic optic neuropathy, or central retinal artery occlusion. In series of GCA patients, 15% developed permanent visual loss, either unilateral (51%), bilateral simultaneous or sequential (30%), and 17% had a visual field defect (36, 49). Premonitory visual symptoms were present in a quarter to three quarters of patients (19, 24, 49).

A third of involved eyes deteriorated within the first 6 days after steroid initiation, 11% of uninvolved eyes deteriorated within a day, whereas only 15% recovered more than two lines of visual acuity, although remaining deeply impaired (50). Overall, the duration of GCA symptoms was shorter in patients with ischemic events (mean 5 weeks from onset) (36). These data suggest that although most patients benefit from early treatment, some could still be at high risk of cranial complications (36). The precise nature of the risk factors remains unclear in these patients (random lesion of critical arteries? persisting inflammation or thrombosis of stenosed artery?).

Amaurosis fugax revealing GCA is a major sign of compromised ophthalmic perfusion: fluorescein fundus angiography confirms sluggish circulation of blood in relation with low arterial pressure. Moreover, relapse or aggravation of visual signs could be related to a fall of prefusion pressure secondary to hypertensive drugs or to a rise in intraocular pressure (51). MRI may demonstrate wall enhancement of ophthalmic arteries (28) and peri-phlebitis (52). Amaurosis fugax rarely relapses after treatment initiation (24).

Doppler exploration of the ophthalmic artery demonstrates various hemodynamic alterations: reversal of flow (without carotid change), turbulent flow between two focal stenoses, increase in vascular resistance (due to vascular bed stenosis), reduced ocular pulse amplitude, or loss of flow. Interestingly, high flow velocity and vascular resistance obtained at onset are associated with clinical progression and visual deterioration after steroid initiation (53). These data suggest that patients who undergo clinical deterioration have more advanced and critical vasculitis lesions at onset than those who 'responded' to steroids (53). During treatment, vascular resistance still increases whereas blood flow decreases, suggesting a residual intimal thickening and wall fibrosis resistant to steroids (or lag before efficacy), even in unaffected eyes (53). An improvement on fluorescein angiography is indicative of clinical recovery (54, 55), suggesting that retinal penumbra may explain early optic impairment at some point. Finally, lessons from optic ischemia point to the major role of hemodynamical mechanisms downstream arterial stenosis/occlusion.

#### 3.3.2 Lessons from GCA-associated limb arteriopathy

Limb claudication is one of the most frequent symptoms, although sometimes remaining poorly symptomatic (56). Vascular limb manifestations accompany or often reveal general symptoms in ~30–40% of patients (56–59). In half of cases, vascular symptoms appear after treatment initiation (56). Local vascular relapses (stenosis) may occur at the end of the treatment (56). Except in rare cases showing dramatic improvement of stenosis, the resolution of symptoms does not generally accompany arteriographic improvement of stenosis and results from the development of artery collaterals (56, 57). Unlike other types of vasculitis, gangrene is not a common feature of GCA (7), which suggests that lesions of the distal bed or emboli are not involved.

### 3.4 Clinical cranial signs and symptoms

Cranial ischemic events in GCA could be defined as transient or irreversible signs. Transient signs are typical of reversible ischemia or insufficient arterial flow: jaw claudication (painful muscles during chewing), amaurosis fugax or blurred vision, and transient ischemic attack (TIA) (Table 3). Irreversible signs are mainly tongue or scalp necrosis, diverse ischemic ophthalmic signs, and stroke. The incidence of ischemic events occurring within 2 weeks after GCA diagnosis, defined as transient and irreversible events, was 73%, whereas severe irreversible events (AION and stroke) occurred in 34% of GCA (60). Carotid and orbital bruits related with flow turbulence and disappearing a few days after steroid initiation were noted early (14, 61, 62).

**Table 3:** Signs and symptoms related with hemodynamic impairment.

**Signs and symptoms**	**Context suggestive of low flow**
Transient ischemic attack (TIA)	Arterial stenosis
Transient or undulating diplopia	Strokes in watershed areas
Amaurosis fugax	Awakening blindness
Transient visual blurring	Rapidly progressive (hours) blindness
Jaw claudication	Positional TIA or amaurosis fugax
Limb claudication	Anti-hypertensive agents
Carotid and orbital bruits	(Relative) failure of antiaggregant/anticoagulants

Headache is the most frequent extracranial symptom (72%). Clinical symptoms of intracranial GCA are the following: unilateral blindness (26%), cerebellar signs (72%), motor impairment (62%) and impaired level of consciousness (49%), and about half of patients died (26). Dementia is always the consequence of relapsing and extensive infarction (63).

Transient or undulating diplopia from third or sixth nerve palsy may occur in more than 10% of GCA cases in relation with oculomotor muscle or nerve ischemia (32, 64), and may be complicated by oculomotor synkinesis or aberrant nerve regeneration, suggesting peripheral nerve involvement [see Reich et al. (64)]. MRI may reveal third nerve enhancement (“check mark sign”) (65).

### 3.5 Meta-analysis of signs associated with ischemic events

Several authors compared groups of ischemic events among GCA patients to delineate a high-risk clinical profile and select patients who might benefit from aggressive treatments (19). Although the available data are highly heterogeneous and mix brain and extra-brain ischemic events, we provide a metanalysis of clinical and paraclinical signs associated with cranial ischemic events including stroke (Table 4, all the results below are findings from the metanalysis, detailed methods and results are provided in Supplementary material 2).

**Table 4:** Signs predicting stroke events.

**Positive association**
Ophthalmic signs (transient or irreversible)
Vascular risk factors (chronic hypertension, diabetes, dyslipidemia, smoking, atrial fibrillation, age at diagnosis)
Positive TAB
Aortitis
**Negative association**
Polymyalgia rheumatica
Constitutional syndrome or fever
Inflammatory systemic response (High CRP or ESR, low hemoglobin)
Scalp tenderness

Transient signs (OR 5.03 [CI 95% 2.81, 9.01], *p* < 10^−5^), mixing amaurosis fugax and TIA, signs of aortic branch lesions, like aortitis (OR 3.09 [1.39, 6.85], *p* = 0.005) and positive TAB (OR 1.69 [1.11, 2.56], *p* = 0.01), are strongly associated with ischemic events. Other signs of cranial involvement, like abnormal TA on physical examination, jaw claudication, and headache, were not significantly associated.

General signs, including constitutional syndrome (OR 0.64 [0.47, 0.88], *p* = 0.005) and fever (OR 0.60 [0.40, 0.91], *p* = 0.01), PMR (OR 0.66 [0.51, 0.85], *p* = 0.001) were all negatively associated. Inflammatory systemic response was also lower in patients with ischemia: CRP (mean difference, MD −29.01 [−42.61, −15.41], *p* < 0.001), ESR (MD −5.70 [−9.71, −1.69], *p* = 0.005), whereas hemoglobin was higher (MD 0.49 [0.19, 0.79], *p* = 0.001). Platelet and albumin levels were not significantly different.

Among vascular risk factors, chronic hypertension was the strongest predictive factor (1.82 [1.38, 2.41], 10^−4^). Unfortunately, specific data (complications, pressure control or drugs administered) were not available, especially at the time of ischemic events. One series found that treatment by anti-hypertensive agents, especially β-adrenergic inhibitors, was associated with a risk of optic ischemic events but not hypertension (66). Since hypertension is a vascular risk factor, it is often suspected that the risk associated with antihypertensive drugs is a confounding effect (13, 32). Nevertheless, we think that antihypertensive drugs may have facilitated hemodynamic mechanisms that, in turn, triggered ischemic events.

Other vascular risk factors were also predictive of ischemic events: diabetes (OR 1.65 [1.15, 2.36], *p* = 0.006), smoking (OR 1.69 [1.06, 2.68], *p* = 0.003), hypercholesterolemia (OR 1.37 [1.02, 1.84], *p* = 0.04), and atrial fibrillation (OR 2.23 [1.20, 4.13], *p* = 0.01). Among vascular risk factors, diabetes mellitus was the only other significant vascular risk factor (OR 1.84 [1.15, 2.94], *p* = 0.01), whereas a history of ischemic heart diseases was not significant. Antiaggregation or anticoagulation before the diagnosis did not provide significant protection, but an interaction with vascular risk factors may mask a protective effect. Although socio-economic deprivation is probably a proxy for higher tobacco exposure and atherosclerotic risk, a study adjusting for common vascular risks found that deprivation could be a major risk factor of ischemic events, and patients who took aspirin before GCA were still at higher risk of ischemic events (67). Finally, GCA in itself is probably an additional vascular risk factor since the cumulative incidence of myocardial infarction and peripheral vascular disease are higher in GCA patients (68).

Ischemic events at onset are strong predictors of future events. Transient cranial ischemic events like jaw claudication, amaurosis fugax, and irreversible visual loss or stroke are strongly associated with the risk of future strokes (15, 19, 36, 38, 54, 60, 69, 70). Ischemic events developing after GCA diagnosis are often recurrences since 21% of them are relapses, but only 4% of GCA without ischemic events developed a recurrence after steroid initiation (19, 50).

### 3.6 Systemic inflammatory response (SIR)

It is widely thought that the risk of cranial ischemic symptoms is inversely proportional to SIR (36, 71). However, the strict definition of inflammation severity remains unclear. If 'strong' SIR is defined by ≥3 parameters in: fever, weight loss, ESR>85 mm/h, or hemoglobin <11 g/dL (36, 71–73), no patients with an ischemic event fulfilled all four positive criteria (36). However, replication is lacking, and this stratification is not used in common practice. Inflammatory parameters (e.g., ESR, CRP, fibrinogen, anemia) could be lower in GCA patients with stroke (4, 36, 74), although mean ESR remains similar in others (5). A series with short delays from GCA onset to diagnosis found similar levels of inflammation in patients with stroke (75). This suggests that ischemic patients may be referred early before the full-blown systemic response (36, 72, 76). Indeed, GCA patients recruited in fast-track clinics displayed lower mean CRP levels, although higher levels were observed in cases with visual loss (77). Multivariate analysis did not confirm a difference in CRP levels between GCA subgroups with stroke or amaurosis (23, 78). Although a promoting role of neo-angiogenesis was proposed (79), inflammatory syndrome probably does not exert any protective role on ischemia (24). Finally, although it is possible that ischemic complications occur in patients with lower inflammatory parameters, the minor biological difference does not add any clinical value at individual level (80). Of note, CRP levels are variably expressed in mg/dL or in mg/L, leading to erroneous underestimation of CRP levels. Normal ESR at onset is possible in <10% of GCA (81), but CRP is far more sensitive to systemic inflammation. At GCA onset, CRP is very rarely normal [below ≤ 2.5% (81, 82)]. It is typically associated with optic signs and rises rapidly within a few days (83). Combined normal CRP and ESR occurs in <1.2% of cases (84). Series of ischemic cases reported low (but abnormal) median CRP at diagnosis (6 mg/L, range 2–28 mg/L)(5), but normal CRP levels were extremely rare in published cases of stroke (85, 86). In our review of GCA-related stroke, 22% cases had CRP below 20 mg/L, but only one patient displayed double-negative CRP/ESR [none in Hayreh et al. (81) and Bonnan and Balley^1^, and 0.8% in Parikh et al. (82)]. In another series, double-negative CRP/ESR was present in 36% (9/25) of patients with stroke and 18% (3/16) of other GCA patients (87). Therefore, although SIR is not always dramatic, completely false negative biology for inflammation is not an uncommon finding at onset in GCA-related stroke.

Severe ischemic GCA patients were found to display a higher prevalence of VEGF polymorphism associated with a reduced circulating level (88), but this finding has not been confirmed until now.

### 3.7 Does pathology give clues to ischemic mechanisms?

**Figure 1:**
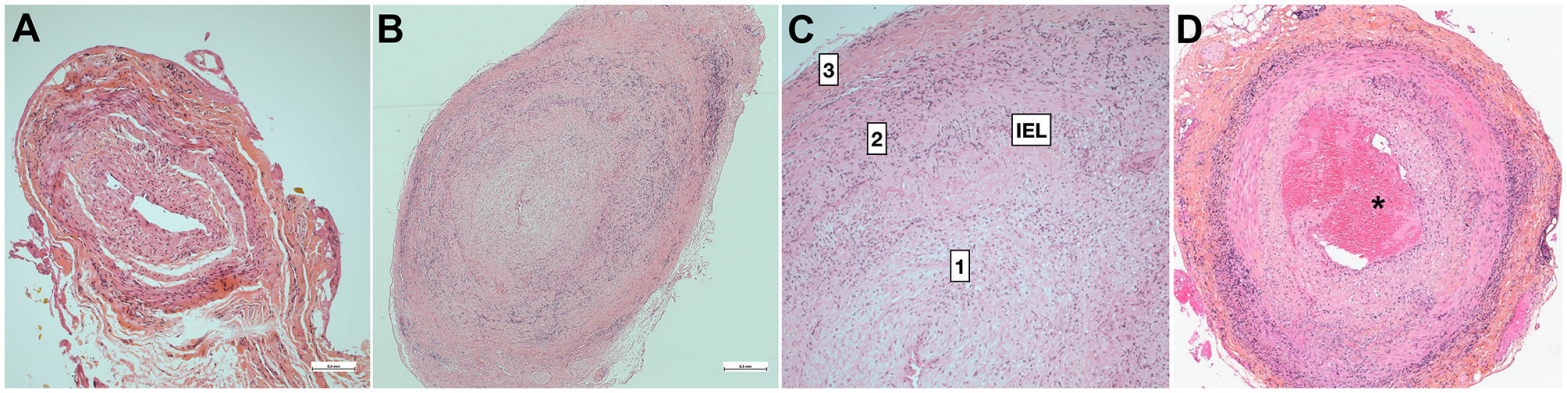
Pathology of temporal artery biopsy. **(A)** False-negative temporal artery biopsy. Minimal non-specific intimal proliferation. **(B)** Positive temporal artery biopsy with full-thickness granulomatous inflammation and slit-like luminal stenosis related with major hyperplasia of the intima [magnified in **(C)]. (D)** Positive TAB with intraluminal thrombus (^*^). 1: intima, 2: media, 3: adventitia. H&E staining. Bar is 0.5mm (Dr Badaro).

GCA is characterized by inflammatory infiltrates of the medium and large vessels, with variable abundance of giant cells and internal elastic lamina (IEL) fragmentation (89) (Figure 1). Random lesions occurring along the arteries are separated by normal tissue (“skip lesions”). Large transmural inflammatory infiltrates correlate (90) or not (91) with higher levels of biological inflammation. The influence of steroids on the main parameters of inflammatory lesions remains modest during the first month (91), whereas healing lesions are observed on biopsies performed more than a year later (92).

Ophthalmic involvement is generally associated with intensity of inflammation, giant cells, and plasmocytes, or with the severity of intimal hyperplasia (93–95). However, the correlation is mostly absent in stroke patients, since none of the histological parameters obtained from TAB (especially intimal proliferation) differed in ischemic groups (90). Luminal thrombosis is observed at the same frequency (95) (Figure 1D). A major limitation is that the temporal arteries do not irrigate the brain or ophthalmic tissues, so focal thrombi found on TAB do not convey any risk of brain or eye embolization. Since the culprit arteries cannot be evidenced except by autopsy, it is uncertain whether the lesions of temporal artery lesions mirror those of the culprit arteries.

One study found a higher ischemic risk in the presence of vascular calcifications (96), but stenosis combining atherosclerotic and arteritis lesions has been rarely described (97). Age-related intimal thickening is aggravated by atherosclerosis (98), but remains moderate and non-occlusive. GCA does not promote atherosclerosis (99), and ischemic events in GCA are not related to complicated atherosclerotic lesions.

Fibrinoid degeneration is always absent in large GCA series (96, 100, 101) and would point to antineutrophil cytoplasmic antibody (ANCA)-associated vasculitides (102). Autopsied cases from long ago reported thrombosis of the coronary, vertebral or basilar arteries infiltrated by arteritis (103, 104). However, detailed pathology was not provided, so it remains unclear whether the mural thrombosis developed on endothelial lesions as arteritis, atheromatous plaque, or an artery stump. Luminal thrombus, which is generally subtle, is observed in <15% of TAB (90, 101, 105), irrespective of steroid treatment (96), and may participate only in luminal occlusion, which is mainly driven by intimal proliferation (95, 101, 106–108) (Figures 1B, C). Reduction of the arterial lumen to slit-like is mostly the consequence of the major intimal proliferation observed in half of the cases (101), and developing until occlusion (101, 109). Moreover, the long-term evolution of intimal hyperplasia after treatment is unclear considering the frequency, extension and degree of involution, although persistence may be variable (110). One case showed a minute focal hemorrhage within the thickened intima (33).

Intimal hyperplasia, which may drive ischemic events, is variable among GCA patients. Delayed treatment, which may lead to an increased burden of lesions, is associated with an increased risk of ischemic events. However, the degree of intimal hyperplasia (on TAB) may be independent from the lag between GCA onset and TAB (91, 94). Since intimal hyperplasia is assessed on a different artery (e.g., TAB) than those involved in ischemic events, its implication may be difficult to confirm (96). We believe that the risk of ischemic events may be related with both the duration of the natural history before treatment, and with each patient's kinetics of reactive intimal hyperplasia. Moreover, persisting intimal hyperplasia in healed patients may contribute to the delayed risk of hemodynamic ischemic events. Intimal hyperplasia is significantly associated with T-cell synthesis of IFNγ and IL1β, and with PDGF-A/B, VEGF and GM-CSF secreted by macrophages and multinucleated giant cells (71, 93), which are high value targets for future treatments.

### 3.8 Explaining GCA selectivity to extradural arteries: putative role of basic structural variations in cranial arteries

It is commonly admitted that GCA lesions target elastic fibers and especially IEL, which is fragmented in the lesions. Why are the intradural arteries spared by GCA? Some authors posit that IEL could be absent in the intracranial arteries, which is erroneous, or that the lower content of elastic fibers might play a protective role, which is contradictory with the persistence of a thick IEL.

#### 3.8.1 Elastic fibers and laminae in extra- or intra-dural arteries

**Figure 2:**
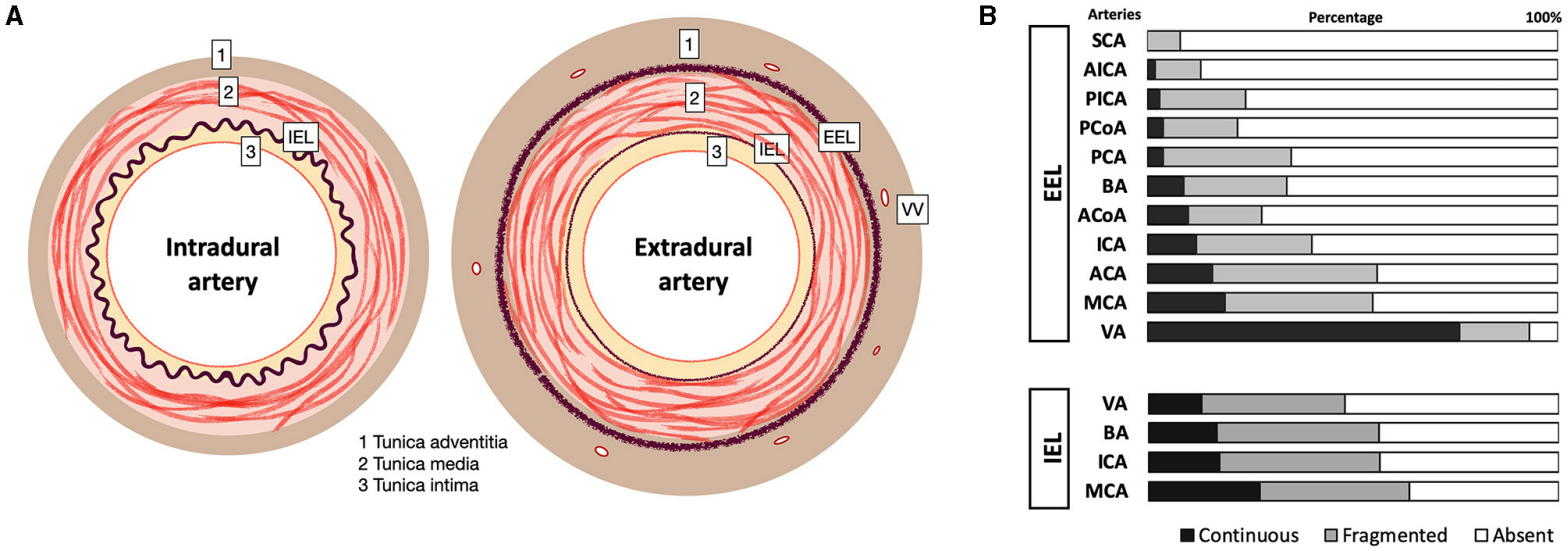
Basic structural variation of intracranial arteries. **(A)** Intradural arteries are characterized by the presence of large internal elastic lamina (IEL), whereas EEL disappear from the entry point into the dura mater. Extradural arteries have prominent EEL and IEL, and elastic fibers in the tunica media and adventitia. In the adventitia, vasa vasorum (VV) surround extradural arteries including the vertebral and carotid arteries, whereas VV are usually absent from the intradural arteries. **(B)** Classically, the transition from extra- to intra-dural artery type occurs in the cavernous portion of the carotid between the posterior (EEL always present) and anterior knees (EEL mostly absent) (113), and within the 5mm of vertebral artery around the dural entry point (111). Although EEL is considered absent from intracranial arteries, structural variations may occur in healthy subjects, and EEL may persist as a continuous or fragmented structure in the intracranial arteries [from Denswil et al. (116)]. ACA, anterior cerebral; ACoA, anterior communicating; AICA, anterior inferior cerebellar; BA, basilar; ICA, internal carotid; MCA, middle cerebral; PCA, posterior cerebral; PCoA, posterior communicating; PICA, posterior inferior cerebellar; SCA, uperior cerebellar; VA, vertebral.

The walls of the cranial arteries are thinner than those of other arteries of the body, owing to thinner adventitial and medial layers (111). Elastic fibers in the tunica media and external elastic lamina (EEL) progressively vanish along the horizontal segment of the cavernous portion of the internal carotid, and within the more distal 5 mm of the intradural vertebral arteries (111–114). Predominance of elastic fibers in the vertebral arteries rather than in the carotids may explain predominance of VA inflammation (115). The discontinuation of EEL is heterogenous (Figure 2), while continuous and even more fragmented EEL may be observed in the intracranial arteries at low frequency (below 20%) beyond the dural entry point (116). In the posterior circulation, continuous EEL is commonly observed in VA and more rarely in the distal arteries (i.e., basilar and anterior inferior/superior cerebellar arteries) (116). EEL disappears in the ophthalmic arteries after penetrating the optic nerve and globe, which correlates with observations of GCA-spared retinal arteries (115).

On the other hand, the thickness of the IEL is mostly unchanged between the extra and intradural segments (111, 113), or even thicker in the brain arteries (114).

#### 3.8.2 Vasa vasorum

Others posited that the absence of intradural vasa vasorum (VV), which might be key structures for initiating inflammation, could be protective (32, 33). At birth, VV are present in the peripheral arteries but not in the brain arteries. During aging, VV develop in the continuity of extradural VV toward the proximal segments of the intradural arteries and are mostly associated with intracranial atherosclerosis (117). In GCA, VV might be the entry point of autoreactive CD4 lymphocytes and VV vasculitis occurring in the adventitia. Consequently, the risk of intracranial GCA lesions may correlate with pre-existing VV (89, 91, 117), which is age-dependent, variable and limited to the proximal segments of brain arteries. Moreover, VV might be more frequent in the intradural vertebral and basilar arteries. Finally, there is probably an individual and age-dependent vulnerability to the proximal intracranial extension of GCA lesions, while the distal intracranial arteries are always spared.

### 3.9 Imaging techniques in GCA-related stroke

Imaging techniques are briefly examined for red flags, tips and pitfalls in the clinical context of GCA-related strokes.

#### 3.9.1 Computed tomography (CT) and CT angiography (CTA)

Thoracic CTA may show aortic dilatation or stenosis of the branches. Severe stenosis and occlusion of the vertebral and internal carotid arteries, but also lesions of branches of the external carotid, are easily observed. Occlusion and inflammation of the temporal arteries appear as a blurred artery wall with perivascular enhancement (*cigar smoke sign*) in 71% of GCA (118).

Increased arterial wall thickness is highly typical of aortitis and may be observed in the smaller branches (see cut-off in Supplementary Table 3). However, CT examination of the cervical artery wall (internal carotid or vertebral arteries) is not as sensitive as other techniques.

#### 3.9.2 Brain MRI

Ischemic brain lesions are not specific and their patterns are discussed elsewhere. Involvement of the frontal branch of the temporal artery is so common that it gave the disease its former name. The cranial arteries emerging from the external carotid, occipital arteries, and the frontal and parietal branches of the temporal arteries are commonly involved. Cranial MRI acquired with protocols dedicated to vessel-wall imaging (i.e., postcontrast fat-saturated 3D-T1-weighted spin-echo acquired on 3T scanner) demonstrate a high proportion of multifocal arterial involvement (119). Arterial lesions display bright contrast enhancement and mural thickening extending to the surrounding tissue of these medium arteries (119), and MRI sensitivity is close to 90% vs ACR criteria, and 100% vs TAB (119). T2-weighted sequences are poorly sensitive to vessel-wall lesions, except in the event of massive inflammation (120). High field 7T MRI confirms the findings and improves the visibility of the small branches of the temporal artery (121).

#### 3.9.3 Ultrasonography (US)

US is an easy-access cost-effective technique which reveals typical signs of GCA, described as an hypoechogenic 'halo' of the arterial wall secondary to the increased intima-media thickness (IMT) that persists during artery compression (122). Cut-off values for IMT may be highly specific and sensitive (~100%) (123) (Supplementary Table 3). Halo sign may be observed in all arteries involved by GCA, especially the vertebral and temporal arteries, and it is rarely influenced by vascular risk factors. US is more sensitive than conventional CT or MRI of the cervical arteries to detect changes. Cases with limited evidence of GCA, e.g., absence of headache and inflammation, can be revealed by this sign, and fast-track management is now based on US screening (12, 41). In patients presenting with a cranial ischemic event, positivity of the temporal artery may be low (29) whereas the vertebral arteries are more frequently involved (124).

#### 3.9.4 PET scan

FDG-PET/CT explores most of the cranial and extracranial arteries involved but is slightly less sensitive than clinical diagnosis (Se 71%, CI 95% [48–89]) or TAB (Se 92%, [62–100]) (125). Positivity correlates with inflammatory parameters (126). Examples are given in Supplementary Figure 3.

It is worthy to note the lower risk of systemic involvement of the large arteries in FDG-PET, especially aortitis in GCA patients suffering from ischemic lesions of the brain and eyes (127–129). The cranial arteries are rarely examined by FDG-PET since assessment of small-diameter arteries was hampered by low resolution in earlier studies. Nowadays, combined FDG-PET/CT allows accurate assessment of the external carotid, temporal, mandibular, vertebral, occipital arteries (130) and of the lower internal carotids, but not their terminations which are too close to the brain. Positivity of one of these arteries is highly indicative of GCA (130). The predictive value of artery FDG-PET positivity for actual or future stenosis or stroke remains unknown.

### 3.10 Drug treatment of GCA-related strokes

In this section, we do not review strategies for treating GCA but focus rather on treatment strategies in the context of GCA-related ischemic events.

#### 3.10.1 Treatment of GCA

High-dose steroid treatment should be started as soon as the diagnosis is made. Fast-track management in specialized units improves delays in diagnosis and earlier treatment decreases the rate of blindness (12, 41, 77). Guidelines for preventing stroke complications are mostly based on retrospective data, but no controlled trial has yet been performed.

Patients with complicated GCA are commonly administered high-dose steroids, either oral (1 mg/kg/d) or IV (15 mg/kg for 1 to 3 days) (4, 131). Megadose or standard oral dose steroids prevented an attack in the contralateral eye but slightly influenced visual outcome in the already affected eye (51). Data obtained in the treatment of visual loss suggest that delayed initiation of steroids (≥24 h after visual loss) dramatically decreases the expected rate of sight improvement (38). Although not fully analyzed, this observation suggests that ophthalmic ischemia could be at least partly reversible (albeit with minimal clinical gain), as a penumbra caused by reduced blood flow may be reversed by an acute effect of steroids on vasculitis. Tapering steroids may trigger GCA relapse, and the patient should receive careful and comprehensive monitoring of potential relapses.

Immunosuppressive drugs are sometimes used early (132). The influence of methotrexate on the risk of future visual loss remains uncertain [no stroke occurred in Hoffman et al. (133)]. An incident stroke was observed in an infliximab-treated group but not in the placebo group (134). Tocilizumab (TCZ), which targets IL6-receptors, can be given in association or as monotherapy to avoid steroid treatment. However, various issues are to be considered in the context of GCA-associated strokes:

a) no patient developed early stroke in the GiACTA trial, so the preventive effect against stroke could not be compared with the effect of steroids (only one stroke and visual loss occurred at day 254) (135), whereas in an observational study, 40% (2 of 5) of TCZ-treated patients (136);
b) worsening of arterial stenosis occurred during TCZ treatment so arterial follow-up should not be neglected (137);
c) although no head-to-head comparison is available, the rate of vision loss occurring after initiation of treatment was low but roughly similar in TCZ- or steroid-treated patients [see Amsler et al. (129)]. The lower incidence of visual complications in patients receiving late add-on TCZ may also illustrate the spontaneous decrease in relapse risk (138);
d) TCZ does not improve acquired vision loss any more than steroids.

Consequently, TCZ is an interesting treatment for GCA, but its benefits (steroid-sparing) related with stroke prevention have not yet been proven.

#### 3.10.2 The so-called resistance to steroids during treatment initiation

Non-compliance with steroid therapy and premature discontinuation due to side-effects occurred in 3 of the 8 patients who suffered early ischemic complications (19). However, stroke developing within the first days after steroids are puzzling and authors discussed several putative causes: arteritis refractory to steroids (139, 140); steroids triggering ischemic events (15, 22, 51, 103, 141–148); insufficient steroid doses (103, 149, 150); or promotion of thrombosis by steroids via effective inhibition of prostacyclin but defective inhibition of platelet thromboxane (151).

Several days of steroid therapy may be needed to halt the inflammatory process, whatever the steroid dose (24, 51). This simple explanation for this is that most of the ischemic events occurring after steroid initiation occur within 5–7 days, especially optic events (51), but are rare thereafter, even though the dose is still high. This pleads against the triggering role of steroids. Consequently, remission (i.e. no subsequent ischemic event) obtained by immunosuppressive drugs given as add-on therapy after a within-a-week relapse is probably more due to natural history than to steroid resistance being overcome by add-on drugs (129, 140).

#### 3.10.3 Treatment of GCA-related stroke

Use of antiplatelets and heparin is proposed empirically at GCA diagnosis (17, 19, 20), and has been strongly recommended since the seminal work of Nesher et al., who reported a 5-fold decreased risk of ischemic events (19, 152). In another study, taking low-dose aspirin and warfarin before GCA was the main difference in patients remaining free of ischemic events (20). However, the protective effect of treatments remains controversial and patients with severe ischemic complications more often receive anti-aggregants or anticoagulants (15, 19, 29, 60). Other studies failed to demonstrate any difference in the incidence of ischemic events between GCA patients previously treated by anti-aggregants or not (4, 16, 29, 153, 154), suggesting a lack of protection, although a protective effect may have been counterbalanced by a higher prevalence of vascular risk factors. Moreover, recurrent TIAs, which are probably related to stenosis, seem resistant to heparin (155). The mechanisms involved in the preventive action of aspirin on GCA-induced stroke are not clearly understood: prevention of embolization from artery to artery (in keeping with the efficiency of warfarin), or the preventive effect of aspirin on intimal hyperplasia via the NF-κB pathway (156), like the action of steroids, or the repression of IFNγ transcription (with no effect on the transcription of IL1β and IL6).

Heparin, warfarin or anticoagulants were used to prevent and treat ischemic complications on the hypothetical basis that thrombosis is promoted by anti-cardiolipin (aCL) antibodies (157). However, aCL are recovered in up to a third of GCA patients, involute within months after steroid treatment, and are not significantly associated with acute ischemic or stenotic events in GCA (19, 158–160).

Anticoagulants might hypothetically prevent thrombo-embolic lesions promoted by severe stenosis or occlusion, although more evidence is still required. Future therapies would ideally combine suppression of inflammation, prevention of thrombosis, and above all should target (both prevent and reverse) luminal occlusion [e.g., imatinib decreases outgrowth of myointimal cells by targeting PDGF (161)].

Since stenosis and occlusion of the cervical arteries are common, these patients are also at high risk of hemodynamic stroke. Flat bed rest is recommended in patients suffering from optic ischemic events and is mandatory to prevent hemodynamic TIA and stroke in high-risk patients.

### 3.11 Transient ischemic attacks (TIA)

TIA and amaurosis fugax are common warning symptoms of GCA. Around 25–50% of patients with amaurosis fugax may develop definitive vision loss, usually within days (38, 69), whereas amaurosis fugax preceded acute vision loss in 30% to 65% of cases (19, 24, 49, 54). TIA may also involve uncommon territories, e.g., lingual artery leading to paroxysmal tongue numbness (14). TIA preceded stroke in only 6% to 23% of GCA-related strokes (19, 36), and also occurred in 2% of uncomplicated GCA (36). However, the frequency of unspecific symptoms like dizziness observed at GCA onset suggests that the incidence of TIA is underestimated. TIA commonly occurs in GCA patients suffering from vertebral or internal carotid artery stenosis (cavernous segment), and may occur (and recur) months before diagnosis (162). TIA commonly disappears following initiation of steroids but may rarely recur (163, 164), probably depending on the reversion or compensation of arterial stenosis (165).

The relationship between TIA in the territory of the stenotic carotid and low blood pressure is rarely questioned. In one case of GCA-related stroke where angioplasty was ruled out, stenosis persisted despite IS therapy and strokes recurred over several months (166), while TIA never recurred in others treated by angioplasty (167). Transient symptoms, especially orthostatic complaints (including dizziness, syncope), commonly occur in patients suffering from severe bilateral vertebral stenosis (168); positional or pressure changes dramatically modify symptoms and perfusion (169–172); persisting decrease in perfusion may reverse after steroids (173); and bright-light amaurosis fugax is a symptom of severe carotid stenosis (171). The GCA literature is mainly focused on irreversible lesions, whereas the influence of hemodynamic triggers on amaurosis fugax, TIA and strokes in watershed areas (WA) has not been systematically examined.

### 3.12 Patterns of lesions: stroke and cervical arteries

#### 3.12.1 Patterns of stroke

Data were collected from a previous review of the literature which analyzed 75 documented CGA-related cases (see text footnote 1).

##### 3.12.1.1 Ischemic lesions predate watershed areas

Given the high rate of stenosis and occlusion involving the carotid or vertebral arteries, WA are expected to be the most common targets of ischemic lesions occurring at onset (Table 5). As expected, all cases of ischemic lesions occurring in WA also present severe stenosis or occlusion of the afferent arteries. In the carotid territory, WA are probably involved in up to 89% of strokes, both sides being involved in half of them (Supplementary Figures 1, 2). Moreover, the difference between transient symptoms and minute ischemic lesions gives strong clues to the hemodynamical mechanisms involved [example in Dimancea et al. (174)]. Recurring perfusion impairment may precede imminent hemodynamic stroke (175).

**Table 5:** Typical ischemic MRI patterns in GCA-related strokes.

	**Typical**	**Atypical**
Anterior (carotid)	Bilateral lesions	Unilateral lesions
	Small focal lesions	Whole artery stroke (MCA or ACA)
	WA (internal > cortical)	Cortical lesion in non-WA
		Lenticulo-striate territories
		Hemorrhage (except hemorrhagic transformation)
Posterior (vertebro-basilar)	Bilateral cerebellar	Isolated complete PCA lesion
	WA cerebellar lobar	Complete cerebellar artery (or wedge-shaped)
	WA MCP	Isolated pontine stroke
	Lateral medullary (one side)	

ACA, anterior cerebral artery; MCA, middle cerebral artery; MCP, middle cerebellar peduncle; PCA, posterior cerebral artery; WA, watershed areas. Proposed from literature review of all reported cases with available brain MRI (see text footnote 1).

WA are more difficult to ascertain in the posterior circulation but could be involved in up to 67% of patients. A typical posterior WA involves one or two sides of the middle cerebellar peduncles (MCP), as was found in a study in which a quarter of patients had a posterior GCA-associated stroke [(43), see text footnote 1]. MCP are very rarely involved in non-GCA strokes (below 0.12% of strokes (176), and among 13 bilateral cases of MCP lesions found in the literature, one suffered from GCA (177, 178). Other WA in cerebellum, pons, or brainstem are difficult to evaluate. The hypothesis of hemodynamic stroke in WA is rarely evoked by authors, so its prevalence remains uncertain.

##### 3.12.1.2 Non-WA are also involved

Ischemic lesions extending to all the arterial territories seem exceptional and mostly involve complete cervical occlusion of the cervical arteries in late or recurring cases. Except in these severe cases, strokes involving an entire arterial territory (e.g., complete MCA, PICA, or pontine paramedian artery) were almost never observed at onset, and stroke involving cerebellar cortex almost never appeared as typical wedge-shaped ischemia. Instead, territory lesions were almost always incomplete, small, multiple, or ill-defined, and often associated with typical WA lesions (179). In fact, a publication bias may have excluded typical territorial stroke patterns from the selected figures. Non-specific small lesions involving the pontine or cerebral peduncle were described, but the mechanism and territories could not be ascertained.

Lesions of the perforating arteries (lenticulostriate) seem very rare (180–182). Curiously, the anterior choroidal artery (AChA) seems always to be spared, since GCA lesions are limited upstream to the extradural carotid, whereas the AChA is a branch of the internal carotid arising downstream near the posterior communicating artery.

Lesions of the lateral medullary may occur and are probably due to the abutting of the artery within the first 5 mm of the intradural part of the vertebral arteries, which are heavily involved by GCA (115). In cases with artery abutment above the end of the vertebral arteritis process, a thrombus may extend upwards from the arteritic segment into the proximal part of the PICA (115). Rare cases of lateral medullary lesions may also occur in patients apparently free of vertebral stenosis, although TOF sequences may reveal an irregular wall (183) and contrast enhancement of the wall provides obvious evidence in some cases (28). These lesions do not have the 'comma-sign' suggestive of medulla WA, which is a linear ischemic lesion located at the medial border of the lateral medullary territory (184). Cases previously treated by aspirin-dipyridamole and coumadin (due to vascular risk factors) may develop stroke in the lateral medullary area (183), thus suggesting an elective arteritis location.

#### 3.12.2 Patterns of involvement of brain-afferent arteries

The common carotid arteries (CCA) were long considered to be spared by GCA (115), and may be used to implant a vascular by-pass (185). However, up to 30% of CCA were involved in a recent series (35). The ophthalmic and ciliary arteries are always involved outside the eyeball and the optic nerve. Classically, the vertebral arteries are severely involved two to three times more often, whereas the internal carotids are mostly spared by GCA (9, 14, 87, 115, 186). Autopsy series highlighted the discrepancy of the less severely affected internal carotid lesions, even in the most involved cavernous segment (9, 97, 115). However, older series based on angiography probably underestimated the prevalence of siphon stenosis (62). In a classical series of 166 TBA-positive GCA collected in 1988, 31% failed to demonstrate involvement of the large arteries, although 19% had permanent or transient ischemic complications (14), which is a very unusual finding in view of the recent literature.

**Figure 3:**
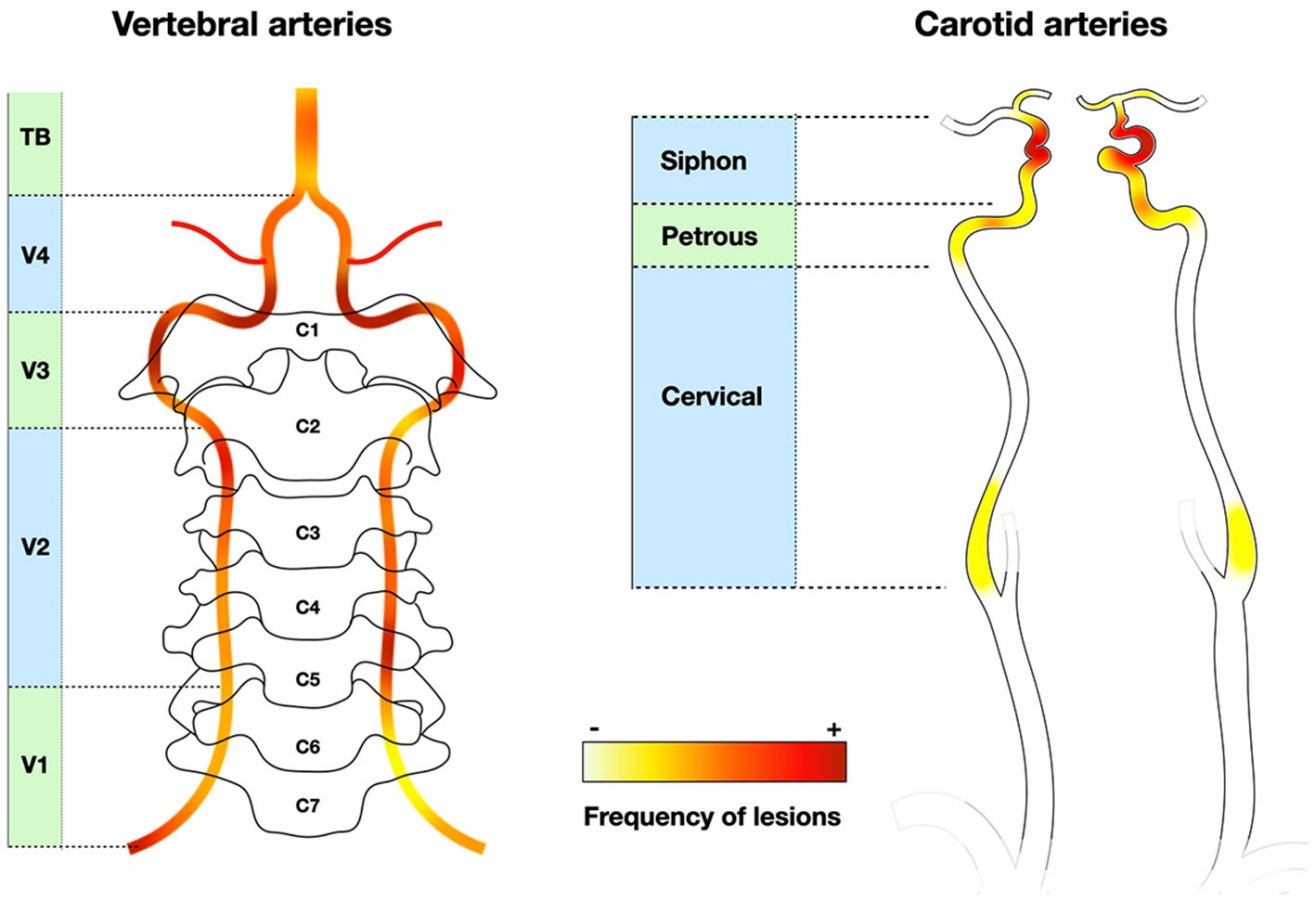
Frequency of stenotic lesions in cervical arteries. Vertebral arteries are often involved, mainly in V3 and V4, whereas internal carotid lesions are located at the level of the siphon. External carotid branches are not depicted. Classically, lesions are localized in V1 and V4 for vertebral arteries (9, 64), or in the entire vertebral and terminal carotid arteries (not represented) (115). Heatmap based on Beuker et al. (87) and Ruegg et al. (188).

In a series of 62 consecutive GCA (non-selected for stroke), the frequency of arteriographic lesions (with ischemic event or not) was 17–21% in each carotid artery and 11–17% in each vertebral artery (187). The prevalence was higher with optimized brain MRI sensitive to mural inflammation: ICA 40%, MCA 3.5%, VA 68%, and BA in none (47). In this systematic brain assessment study, up to 15% of GCA patients presented with stroke, all of them displaying cervical artery involvement (47), which is far higher than the usual incidence of clinical stroke. In a series of GCA-related stroke, the frequency of artery lesions is even higher: ICA 48%, MCA 19%, VA 84% (stenosis V3 and V4 segments), BA 16% (87) (Figure 3). Therefore, the frequency of cervical artery involvement was probably largely underestimated in earlier studies.

The modality of artery assessment strongly influences the association with the risk of ischemic complications: with FDG-PET, the prevalence of carotid or vertebral artery involvement was similar between groups, whereas exams sensitive to luminal stenosis/occlusion demonstrated more frequent lesions in ischemic patients (124). The discrepancy between apparently moderate carotid stenosis and perfusion deficit with bilateral stroke in WA is sometimes surprising (189), again underlining the risk of underestimation due to short stenosis of the ill-depicted tortuous carotid siphon.

#### 3.12.3 Carotid and vertebral artery lesions cluster separately

A tree dendrogram algorithm classifies carotid and vertebral involvement in very different subgroups of patients: the former with mesenteric and aortic lesions, the latter with renal and iliofemoral lesions (187). Our review of stroke patients also points to a separation between anterior and posterior stroke, since fewer than 15% of cases involved both anterior and posterior circulation (at least at onset) (see text footnote 1), but data concerning the other arteries were lacking. Tandem lesions of the arteries (e.g., both carotids, or both vertebral arteries) are common, and bilateral infarction occurred in half of the cases (87). No explanation yet accounts for these associations. There might also be a clustering of patients prone to ischemic complications (190). This hypothesis was proposed by Font et al. (49) to explain visual loss after steroid initiation by exclusive determinism, although alternative explanations are possible.

#### 3.12.4 GCA-related stroke without apparent artery lesions

Sometimes, no stenosis is reported yet it cannot be ruled out (28, 145, 191) (#8). Nevertheless, cases of strokes with parietal inflammation but no apparent stenosis of the vertebral arteries have been reported. For example, a case of minute stroke in the area postrema was associated only with a vertebral halo sign but no stenosis (192). Other reports describe cases of transient vertebrobasilar attacks or Wallenberg syndrome with vertebral artery inflammation and no stenosis (79, 131#3, 178).

Although rarely reported, cases of GCA-related stroke might occur without apparent stenosis at the time of the stroke. In these cases, one may suppose the selective ostial involvement of small arteries (perforating or PICA) abutting to lesioned vertebral/basilar arteries.

#### 3.12.5 GCA is not a form of brain angiitis, except in rare cases

Cases involving the vertebral segment V4 below the basilar artery [e.g. (33)] or the few millimeters of the intradural carotid siphon were excluded from the definition of brain angiitis. A diagnosis of *brain angiitis* was sometimes offered to explain stroke occurring in patients with stable extra-cranial lesions and intra-dural lesions of the vertebral/carotid arteries (132). Moreover, the causal role of GCA-related *brain angiitis* is often put forward as a possible cause of stroke, but without providing any reference to unequivocal cases. The term should be avoided since it supposes the implicit and unconfirmed expectation of vasculitis of the medium and small arteries of the brain. No proof of small-vessel arteritis is found in autopsy cases (9, 115), and cuffing of rare peri-venous lymphocytes may reflect a reactive inflammation, as observed in ischemic brain tissue (obtained at autopsy), rather than arteritis (193). Only very rare cases definitively involved proximal MCA stenosis up to the M2 bifurcation (175, 194–197), in which reversiblity of luminal stenosis was in favor of the hypothesis of GCA. However, in the absence of pathological examination, other reversible or non-reversible mechanisms (vasospasms or atheroma) could also be evoked. At pathological examination, the basilar arteries are sometimes involved (198, 199), as well as the leptomeningeal arteries (198). To our knowledge, the cerebral arteries are exceptionally involved in their proximal segment (9) and are always spared distally from their first segment, so stenosis of the cerebral arteries merely reflects associated intracranial atheroma. Severe stroke does not necessarily mean cerebral arteritis but rather reflects proximal cervical occlusion. Autopsy of a steroid-resistant case with progressive occlusion of the vertebral and carotid arteries and diffuse brain stroke failed to demonstrate any thrombosis in the brain and cerebellar arteries (200).

Although non-GCA granulomatous arteritis of the small brain arteries (i.e., primary arteritis of the CNS or PACNS, or Aβ-related amyloid angiitis) is now clearly documented, it was long confounded by the old nosology, and it may still mask stroke mechanisms associated with GCA (33). Interestingly, rare patients with atypical signs suggesting granulomatous brain angiitis were also those younger than 50, and/or having granulomatous lesions restricted to the intracranial vessels or meningitis (see Supplementary Table 5), which are all red flags against GCA. Rarely, GCA may reveal ANCA-associated vasculitides although ischemic brain lesions are not observed in these cases (102).

Pachymeningitis is rare and dural biopsy may reveal typical inflammation in the small dural arteries, since the dura is vascularized by branches of the external carotid (201).

Cerebrospinal fluid (CSF) is usually normal in GCA (202), and CSF WBC count never exceeds 20 cells/mm^3^ (33, 132). In a series of 7 GCA-related stroke, one displayed pleocytosis and leukocyte subsets were normal in all of them (87). Finally, meningitis is mostly associated with a differential diagnosis, especially PACNS (203, 204).

### 3.13 Follow-up and endovascular treatment of arterial lesions

#### 3.13.1 Follow-up

The wall thickness of the aorta and its branches is often increased by inflammation, and contrast enhancement is observed. Follow-up evaluation at 1 year after treatment demonstrates persistence of abnormal wall thickness: the ascending aorta is abnormal in 69% at onset and 43% at year one, while the carotid arteries in 31% at onset and 11% after 1 year (91). Since contrast enhancement is systematically cleared, persistent mural thickening probably involves residual fibrosis and intimal proliferation (91).

At the cervical level, stenosis apparent on angiography may mask the full extent of arterial lesions, whereas mural enhancement may reveal GCA lesions in arteries of apparently normal caliber (26, 166). Inflammatory lesions progressively abate over time. Artery wall enhancement decreases as soon as day two of treatment (205, 206), and MRI sensitivity decreases after day 10 (207). In the cranial arteries, most of the inflammatory signs had disappeared at follow-up several months later (208). On the other hand, a third of the aortic branches (including the common carotid) still show unchanged enhancement, even in TCZ-treated patients (209). Wall enhancement is not always associated with stenosis at onset (166), and its value in predicting future arterial stenosis is unknown. Late persistence of wall enhancement in a segment undergoing stenosis suggests the incomplete control of GCA (33, 210, 211). On ultrasonography, arterial halo persists at day 7 but disappears before week 3 (212, 213). However, an unexpected pattern of change may be observed in the temporal arteries but not in the axillary or subclavian arteries: a sharp decrease in intima-media thickness occurred first in TCZ-treated patients, followed by a transient but small increase to baseline around week 4, then a decrease to a lower thickness (147, 214). If the pattern of change were to be similar in the brain or ocular arteries, this paradoxical transient small change in volume occurring in treated patients might dramatically impede an already reduced blood flow and trigger hemodynamic stroke. To our knowledge, the hypothesis that treatment may transiently expand lesion burden and trigger ischemic events has never been investigated.

The late outcome of stenosis has never been systematically followed up, and uncertainties exist about rates of outcome. Stenosis or occlusion may persist after treatment initiation (132, 166, 167, 215–217) or variably regress, from partial change (30, 46, 168, 218), to complete re-permeabilization (173, 194, 219–223). However, stenosis sometimes appears or worsens after steroid and/or TCZ initiation (44, 86, 137, 166, 200).

Moreover, stroke occurring late after initiation of treatment may not be directly due to GCA but rather to severe arterial stenosis (22, 167). Beyond a persisting stenotic scar, it is unclear whether stenosis can also exacerbate due, its own dynamics in the absence of focal small-grade GCA recurrence. Persistent occlusion may be associated with pre-existing confirmed atherosclerosis (103, 215, 224). One case of GCA-related stroke treated only by antiplatelets demonstrated spontaneous re-opening of an occluded vertebral artery during follow-up in the absence of steroid treatment (27). In brief, systematic follow-up of a known stenosis is very important if a perfusion deficit remains in its territory (189).

#### 3.13.2 Endovascular treatment of stenotic cranial arteries

Since the action of steroids may be delayed or incomplete, surgical endarterectomy (185) followed by endovascular therapy was proposed to overcome threatening stenosis. In a short series of limb artery stenosis receiving balloon angioplasty, immediate success was obtained although moderate artery dissection occurred in up to 40% of procedures, and late restenosis was limited to previously affected areas in association with the biological recurrence of GCA (59). Balloon dilatation was performed in a few patients, sometimes on multiple arteries and/or several times in the same subjects (166, 175, 225). Dilatation of severe stenosis immediately improved the clinical signs (200, 217). Post-catheter dissection occurred in two cases (225).

To our knowledge, stents are rarely used to relieve GCA-related ischemia in the limbs, bowels or heart (58, 190, 226, 227), and since the coronaries are mostly spared by GCA, most procedures involve the cranial arteries. They were mostly used to treat severe relapsing stroke and had a variable outcome: one patient suffering from a rapid progressive course was offered a stent and partial remission was obtained, whereas two others died due to procedural complications (87). Patients with recurrent hemodynamic stroke in WA received distal ICA or VA stenting and improved without relapse [(26, 139, 164, 167, 177, 218, 228, 229)#2]. Another received a superficial temporal artery to MCA bypass and encephalo-myosynangiosis to improve MCA blood flow in the setting of persisting severe carotid stenosis (165). A carotid stent was also used to treat a putative atherothrombotic stenosis re-occluded within months in a patient with occult GCA that had gone untreated (110). Stroke recurred in another patient despite steroids at day 7 due severe carotid stenosis: although the patient received balloon angioplasty, another relapse occurred in the WA within weeks due to restenosis and poor MCA perfusion (175).

Finally, procedures are increasingly being used to treat severe symptomatic stenosis. Considering the high risk of procedural complications due to the frequent location of the stenosis in the clinoid segment, the indications for intracranial carotid stent placement to target high-risk patients with hemodynamic stroke are restricted (Table 6).

**Table 6:** Indication for stent placement [from Biondi et al. (230) and Dawson et al. (231)].

**Clinical failure of medical treatment**
**—recurrent TIA**
**—fluctuating neurological signs**
**—neurological deterioration)**
Hemodynamic stroke with severe stenosis or occlusion, and reduced parenchymal perfusion
Contralateral carotid stenosis or occlusion.

### 3.14 Non-stenotic mechanisms of stroke

#### 3.14.1 No GCA-induced microemboli?

Microemboli generated by artery wall lesions or a hypercoagulable state were postulated to account for brain and eye GCA-related ischemic lesions, although the evidence is insufficient. Acute central retinal artery occlusion (CRAO) due to an embolic mechanism is classically associated with sonographic visualization of emboli in the retinal circulation (the '*spot sign'*), although this sign was not observed in GCA-related CRAO (232), thus ruling out an embolic mechanism. Cotton-wool spots on the retina are usually associated with vasculitis but small retinal vessels are spared by GCA. The authors hypothesized that microemboli may have given rise to these lesions (233), but direct proof was lacking.

A single study detected microembolic signals by transcranial doppler in the ophthalmic and cranial arteries in association with GCA (234). Systematic investigation of microemboli in GCA has not yet been undertaken, so unless the finding is reproduced, their causal role in WA infarction or multifocal brain lesions seems unlikely, since their location and size are not characteristic of microembolic infarction in the distal arterial bed (235). Moreover, microemboli related with atheroma seems to a play only a minor role, if any, in downstream ischemic watershed brain lesions, which are mostly related with the degree of stenosis.

#### 3.14.2 Arguments supporting artery-to-artery macro-emboli

Thrombosis is common in PACNS since the intima is involved by inflammation, but it differs from GCA pathology in which luminal thrombosis is not a common feature (236). Although no proof is yet available, the release of thrombi from inflamed vessels and an embolic mechanism very likely account for GCA-related stroke, probably due to the high causal expectation (4, 21, 191).

Unlike stenosis, mural thrombi are uncommon radiological features of GCA and rare cases have been reported. There was a unique case of a cervical artery lined by multiple mural thrombi that disappeared within days after anticoagulation (237). In another case of a probable GCA, multiple thrombi were observed in the ascending aorta (238), and a third case was observed during autopsy (106). Cases of coronary thrombosis may involve multiple mechanisms (239, 240). Cases of GCA-related limb ischemia are related with large artery occlusion, but embolization is not a common mechanism in this setting. One acute case explored by a Fogarty catheter was finally explained by humeral stenosis relieved by angioplasty, but no thrombotic material was evidenced (58). Such a rarity of reported mural thrombi is even more surprising in the era of widespread CTA imaging.

Thrombosis of the brain arteries has rarely been observed during autopsy (198, 241), sometimes extending from an occluded vertebral artery downwards to PICA (141, 144, 242), and is thought to cause distal embolic stroke in PCA (106, 115). Data from autopsy cases may be biased toward advanced thrombotic states, which may not reflect the mechanisms involved in early ischemic lesions. Thrombi were also found in the retinal artery of a GCA patient with visual loss but no apparent local arteritis (97, 243). The structure and composition of thrombi have never been the subject of scrutiny, except for fibrinous thrombi observed in a unique autopsy case (198). Of note, although they were hypothesized long ago (10), hemodynamic mechanisms were ruled out by authors who considered that the cause was embolic and did not envision alternative mechanisms (115).

Finally, thrombi may occur in GCA as infrequent findings. Apart from typical expected changes related with GCA, post-mortem findings in severely affected patients revealed an organizing mural thrombus in the stump of the carotid arteries (115, 244, 245). Clotting probably reflects blood stasis downstream arterial occlusion, and two hypothetical mechanisms can be proposed: (a) migration of stasis thrombus located downstream a thrombosed carotid or vertebral artery (stump syndrome); (b) migration of stasis thrombus located upstream the arterial occlusion, which may migrate after the blood flow restoration due to the steroid-induced resolution of wall inflammation. This hypothesis may account for stroke occurring early after steroid initiation in patients without anticoagulation (246), or for bilateral PCA thrombosis in bilateral occlusion of VA (106). It would be appealing to monitor the natural history of changes occurring in occluded arteries (kinetics of repermeabilization, presence of mural/luminal thrombi, etc.).

#### 3.14.3 Improbable stroke mechanisms and brain vessel complications, especially vasospasm

Unlike Takayasu arteritis, steal phenomenon remains rather elusive. To our knowledge, no systematic doppler study of Willis circle circulation has been undertaken in GCA, the perfusion consequences of severe stenosis remain to be established in these cases, and steal phenomenon in GCA is still undemonstrated, although advocated (21, 193, 247).

Venous phlebitis and pulmonary embolism are common complications of GCA that occur more frequently than in the general population (248), but cerebral venous thrombosis does not occur in association with GCA.

Vasospasm has been posited to account for transient lesions and AION, although convincing data were lacking (249). A patient with normal brain MRI 2 days before demonstrated new severe carotid and MCA stenosis, but incomplete resolution of the symptoms suggested an explanation other than vasospasm (195). Two patients suffering from amaurosis fugax presented transient focal stenosis of the retinal arteries (195), but the diagnosis of stenosis may be explained by orthostatic factors (250). Others attributed amaurosis fugax or unexpected visual recovery after AION to vasospasm (51, 249), although hemodynamic factors may have been involved. Headache associated with GCA is described as dull and continuous, but icepick headache compatible with vasospasm has sometimes been proposed despite the absence of imaging evidence (251, 252). We did not find any definitive evidence that vasospasm can trigger stenosis or strokes in GCA, although the issue remains to be investigated thoroughly.

Apart from secondary hemorrhage in the supra-tentorial ischemic areas, spontaneous brain hemorrhage is very exceptional and its cumulative incidence is similar in GCA with paired controls (253). A complete review in 1988 found only two cases (14), and we found none in our review. This is an important point distinguishing GCA from other types of brain arteritis.

Unlike the aorta, the cervical and brain arteries are not prone to GCA-related aneurysm, except for an isolated case of intracranial aneurysm in the distal carotid (231).

#### 3.14.4 GCA-induced dissection of the cervical arteries: fact or pitfall?

While aortic dissection may complicate GCA, dissection of the cervical arteries requires caution. Segmental wall involvement may progressively lead to a transition from normal to stenotic segments, i.e. the *slope sign*, extending over a long arterial segment and mimicking artery dissection (87, 254). Wall thickening with or without stenosis should lead the clinician to suspect artery “dissection” (28, 45, 255–260), but general signs, old age, and the absence of high signal or the presence of gadolinium enhancement on T1-weighted images are in favor of a diagnosis of GCA. Images showing dissection of the cervical arteries are not typical and specific high-signal wall lesions in fat-saturated T1-weighted are absent, so this purported dissection may finally be explained by inflammatory wall infiltration. Severe ptosis related to third cranial nerve palsy homolateral to a carotid lesion may suggest Horner syndrome (260). Pathological autopsy confirmation of vertebral dissection was obtained twice, but one patient had already undergone salvage angioplasty before (257), which is also a risk factor for dissection. However, a cleavage plane cut appeared devoid of erythrocytes (104, 257), which may suggest an artifact commonly observed with GCA lesions [e.g., (33, 105)]. Finally, although GCA-related cervical dissection may occur, GCA may mimic cervical dissection.

### 3.15 Early and late stroke recurrence are GCA-related and -unrelated

#### 3.15.1 Early recurrence

Stroke recurrence may indicate incomplete control of GCA shortly after treatment initiation, a relapse of GCA during steroid tapering, the natural history of persisting severe stenosis, or an unrelated event in elderly patients (3, 189) (Table 7). Two periods are vulnerable to stroke recurrence (19): within days after steroid initiation, and months later during steroid tapering. Patients should be clearly briefed before steroid tapering so that they immediately report any general or even minor sign, in order to avoid delayed treatment and GCA-related stroke relapse.

**Table 7:** Possible mechanisms of GCA-related TIA and stroke.

**Mechanisms**	**Before treatment**	**Within week post drug initiation**	**Later than week one**	**Late (years)**
Delayed steroid efficiency	N/A	+++	-	-
Resistance to steroids	N/A	(+)	(+)	-
GCA relapse on steroid tapering	N/A	-	+++	+/-
Persistent stenosis or occlusion^a^	+++	++	++	+/-
New stenosis or occlusion	+++	++	++	-
Thromboembolism (from mural inflammation)	+	+	(?)	-
Unrelated mechanisms	+/-	+/-	+/-	++

^a^hemodynamic stroke in WA.

Recurrent strokes were observed in 8% to 38% of patients in some series (19, 87), suggesting that there is a subset of patients with a rapidly progressive course (87). However, most strokes occur before therapy onset or immediately after, and they occur much more frequently in the vertebrobasilar territories than in the carotid ones (15). Stroke recurrence may occur after treatment initiation although stenotic lesions apparently improve on control exams (193), raising the question of a focal low-grade relapse or unrelated cause. Early initiation of drugs (including cyclophosphamide or TCZ) failed to suppress stroke recurrence (87, 136), reinforcing the hypothesis that GCA relapses do not completely account for ischemic relapses.

#### 3.15.2 Late recurrence

Stroke occurring later than the first month after treatment initiation raises specific problems and should be carefully scrutinized: failure of steroid tapering requiring treatment intensification, decompensation of persistent, worsening or resistant stenosis, or expression of unrelated vascular risk factors? Once treated, GCA patients have only a slightly higher risk of stroke than controls, but patients with stroke have a higher risk of GCA relapse (6). The prevalence of GCA relapses among cases of late stroke is unknown, but many authors consider the latter to be co-extensive of GCA relapse, although biological parameters of inflammation may remain low and precise exploration of arteries (i.e., relapse of local parietal inflammation on MRI or FDG-Pet) is mostly unreported in the literature. A woeful diagnosis of '*brain vasculitis'* mechanically leads to treatment intensification, even though the mechanisms triggering stroke remain uncertain (132).

Recurrent carotid stroke in WA has been reported in association with persisting stenosis of the terminal carotid despite aggressive treatment (110). Autopsy demonstrated that the stenotic segment involved in these late relapsing cases of stroke displayed only fibrosis, re-permeabilization, and fragmentation of the IEL, but no active arteritis. This rare finding supports the hypothesis of a “*burned-out vasculitis”*, i.e., a sequel of former arteritis as observed in Takayasu arteritis. Therefore, the real challenge is to distinguish the persistence of stenotic arterial scars from active arteritis resistant to steroids and/or the required intensification of immunosuppression. In practical terms, confirmation of active (resistant) GCA or residual intimal fibrosis is technically undetermined, although wall enhancement on MRI may be of major interest. Stroke relapsing in the same arterial territory vascularized by a sub-occlusive artery merely suggests hemodynamic impairment, and immediate improvement is expected after revascularization (217).

Reduction of the lumen caliber of the cervical arteries by intimal proliferation mostly persists after treatment. Early GCA and its relapses may produce a stepwise reduction of “*luminal reserve”*until the hemodynamic consequences trigger stroke. Therefore, early treatment of GCA may prevent the occurrence of acute events, preserve the luminal reserve and reduce the risk of late ischemic events. Luminal reserve may also account for the predominance of stroke in territories vascularized by the vertebral arteries rather than the carotid ones.

#### 3.15.3 Criteria for GCA-related and -unrelated stroke: late strokes are often unrelated

Diagnosing GCA-unrelated stroke poses problem since the definitions of GCA-related stroke are subjective: (a) “*occurrence close to GCA onset”*; (b) up to 2 or 4 weeks after steroid initiation; (c) in the absence of obvious vascular risk factor or atrial fibrillation (Supplementary material 2, Table 1). Some cases occurring around GCA onset and during follow-up are unrelated with GCA. Although difficult to ascertain especially around GCA onset, up to a quarter of incident cases could be GCA-unrelated during the first year (262), and a similar cumulative incidence of stroke with paired controls suggests that subsequent cases are mainly unrelated (253). We found no data (e.g., ischemic pattern or clinical signs) that could help to identify GCA-unrelated strokes occurring during follow-up. It is noteworthy that only one case of stroke (196) reported in the literature was considered eligible for thrombectomy, suggesting that the real percentage of GCA-related stroke at onset could be underestimated in the presence of obvious risk factors like atrial fibrillation or atheromatosis. Moreover, a GCA-related general inflammatory state may increase the risk of an embolic event, and a mural thrombus due to cardiac stroke may occur (263).

**Figure 4:**
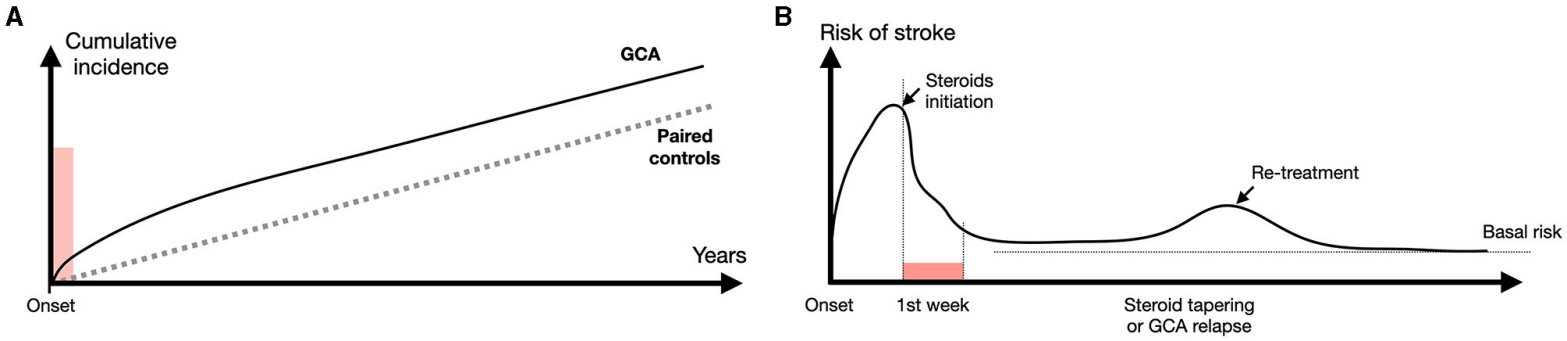
Risk of stroke during follow-up of GCA patients. **(A)** Overall risk of stroke in GCA patients compared with paired controls [from Tomasson et al. (68), Lo Gullo et al. (253) and Amiri et al. (261)]. A slightly higher incidence of stroke is observed within weeks after treatment initiation: thereafter the overall incidence remains roughly similar with paired controls. **(B)** The higher risk of ischemic event precedes treatment initiation, which is followed by a drop close to basal level within a week. Risk slightly increases during treatment tapering and/or GCA relapses. However, this incidence remains low compared with unrelated strokes.

GCA patients also share the age-risk of stroke (Figure 4). Long-term follow-up confirmed new ischemic events in up to 40% of GCA patients at month 160 (39). At 10 years follow-up, studies based on large series of control-matched GCA confirmed a similar risk of combined ischemic events, TIA, and ischemic or hemorrhagic stroke, although amaurosis fugax was more frequent in former GCA patients (68, 253), suggesting persisting compromised vascularization. The rate of atherosclerotic change does not seem to be increased in former GCA patients (99).

Recurrence of inflammation may be observed during relapses, but GCA is frequently self-limiting thereafter for over a year (7). Therefore, the recurrence of GCA or a new arterial lesion is highly improbable after 1 or 2 years. Extended follow-up over several years demonstrates that late strokes predominate in the carotid territory in the absence of GCA relapse (15, 39), suggesting that most cases are unrelated with previous GCA.

## 4 Conclusion

**Figure 5:**
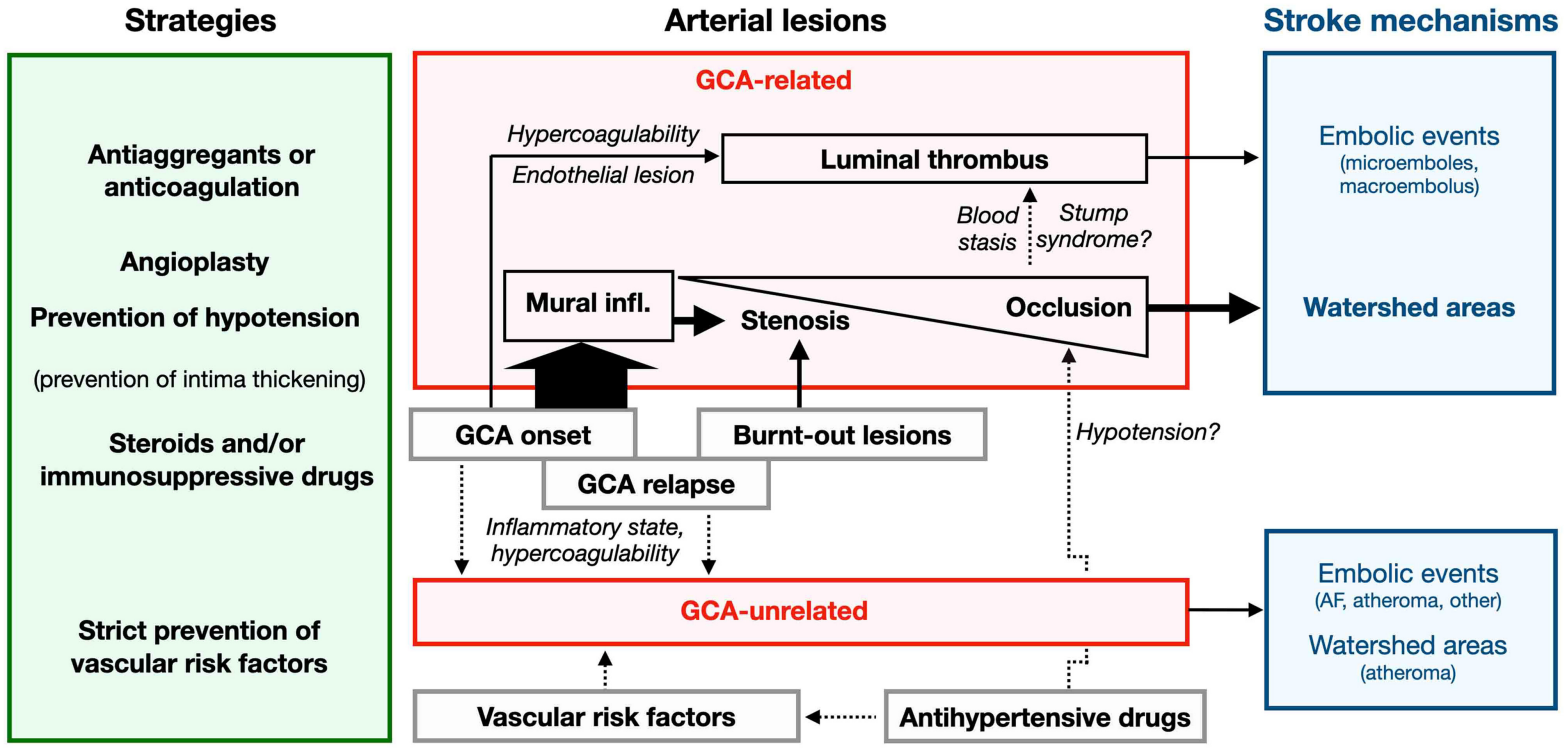
Proposed GCA-related stroke mechanisms and treatment strategies.

Ischemic complications of GCA are often attributed to arteritis without any further thought, yet this is a shortcut avoiding a real investigation into the various mechanisms of stroke. This review demonstrates that this simplistic explanation is inadequate and that specific mechanisms may be involved (Figure 5). First, proof of clotting or an arterial embolism is mentioned in passing more often than it is confirmed. On the other hand, the typical clinical features of GCA-related ischemic complications, i.e. amaurosis fugax, rapidly progressive blindness, TIA and strokes in WA, fit well with the hemodynamic mechanisms related to stenosis/occlusion. The rule of thumb for treatment should be to restore the inner arterial caliber as soon as possible. Steroids should be initiated as early as possible, although they are fully efficient on the inner arterial caliber probably only after several days. Aspirin is recommended to prevent stroke although its preventive efficiency remains modest. Drugs are efficient to control general symptoms and biological parameters in GCA, but their results do not always match with imaging features, especially stenosis. In a context of severe arterial stenosis, the best preventive strategy should be to attenuate the local inflammation and to release the obstacle to blood flow.

Finally, GCA-related strokes are most due to a hemodynamic mechanism restricted to patients with major wall lesions. Nowadays, strokes have become rare events and the early diagnosis of GCA is probably the best way to prevent these severe complications, as is already the case in the prevention of ophthalmic ischemia.

## Author contributions

MB: Conceptualization, Methodology, Writing—original draft, Writing—review & editing. SD: Formal analysis, Methodology, Software, Writing—review & editing.

## Footnotes

^1^Bonnan M, Balley G. *Stroke Pattern in Giant-Cell Arteritis Mostly Involves Watershed Areas*. (2022).
