# Supplementary material for: Giant-cell arteritis related strokes: scoping review of mechanisms and rethinking treatment strategy?
Source: Front Neurol. 2023 Dec 7;14:1305093. doi: 10.3389/fneur.2023.1305093 (PMC10733536; doi:10.3389/fneur.2023.1305093)
Supplement: Supplementary file 1 [file Data_Sheet_1.pdf]

Supplementary Material 1

Giant-cell arteritis-related strokes: scoping review of mechanisms and rethinking treatment strategy?

Mickael, BONNAN*, Stéphane, DEBEUGNY

*** Correspondence:** Corresponding Author: mickael_bonnan@yahoo.fr

1 Supplementary Data. PRISMA Flow Diagram.

MEDLINE database was searched up to May 2023. The search strategy was designed to broadly include vascular complications. PubMed search: *("giant-cell arteritis"* OR "*temporal arteritis*") AND (*stroke* OR *brain* OR *cerebral* OR *cerebellar* OR *carotid* OR *basilar* OR *vertebral* OR *watershed*)*"*.

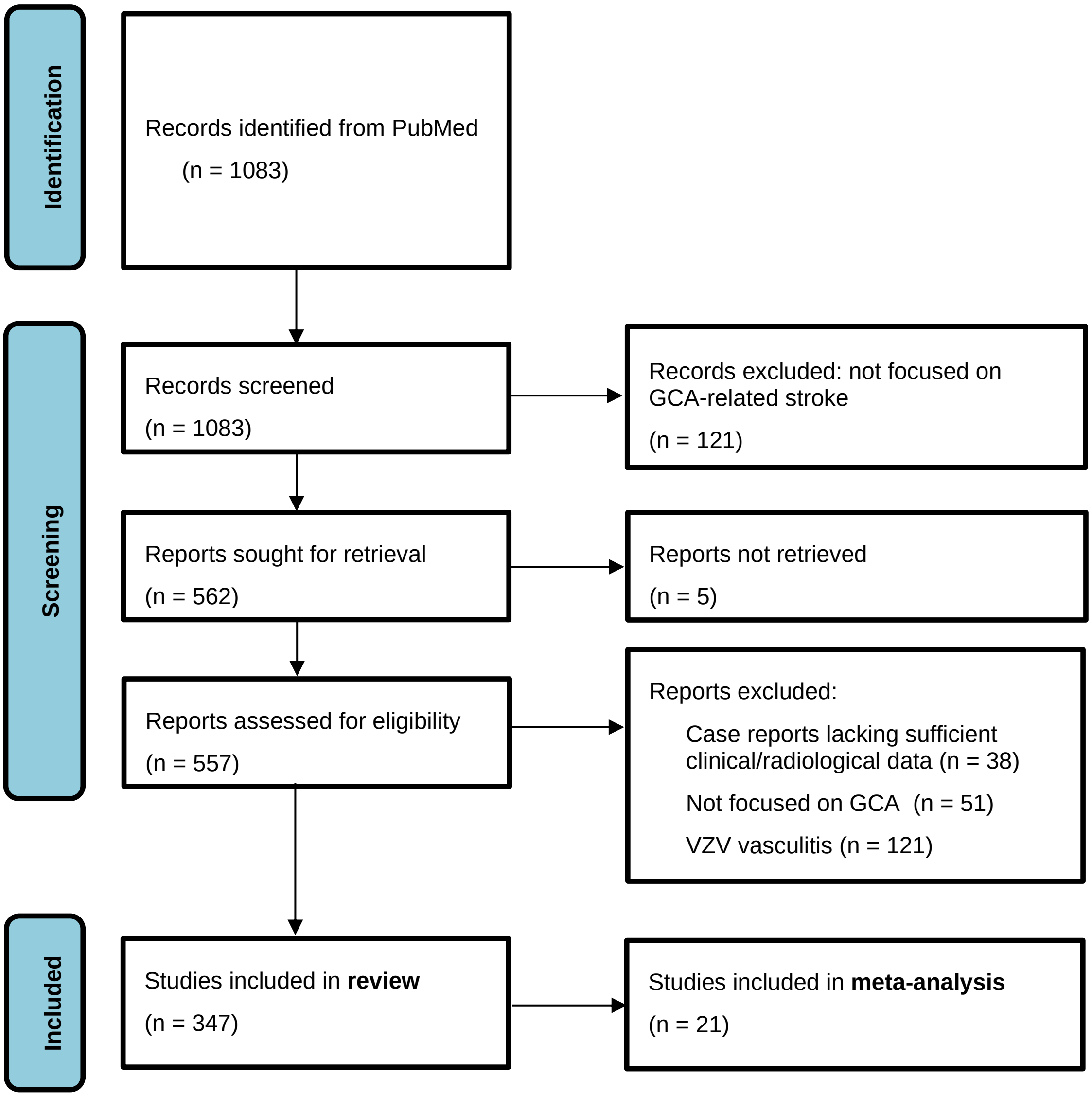

2 Supplementary Tables

2.1 Supplementary Table 1. American College of Rheumatology (ACR) 1990 criteria (Hunder et al., 1990).

≥3 of the following criteria:<br>• Age at least 50 years at disease onset<br>• New onset of localized headache<br>• Temporal artery tenderness or decreased temporal artery pulse<br>• Elevated erythrocyte sedimentation rate (≥50 mm/h)<br>• Arterial biopsy showing necrotizing arteritis characterized by predominance of mononuclear cell infiltrates or granulomatous inflammation with multinucleated giant cells

2.2 Supplementary Table 2. Classification criteria for GCA (Ponte et al., 2022): 2022 American College of Rheumatology / EULAR.

**Absolute Requirement**	
Age ≥50 years at time of diagnosis	
**Additional clinical criteria**	
Morning stiffness in shoulder / neck	**+2**
Sudden visual loss	**+3**
Jaw or tongue claudication	**+2**
New temporal headache	**+2**
Scalp tenderness	**+2**
Abnormal examination of the temporal artery	**+2**
**Laboratory, imaging, and biopsy criteria**	
Maximum ESR ≥50mm/hour or max. CRP ≥10mg/Liter[a]	**+3**
Positive temporal artery biopsy or halo sign on temporal artery ultrasound	**+5**
Bilateral axillary involvement[b]	**+2**
FDG-PET activity throughout aorta	**+2**
**A score of ≥6 points is needed for the diagnosis of GCA**	

[a] prior therapy initiation
[b] defined as luminal damage (stenosis, occlusion, or aneurysm) on angiography (CT, US, MR, FDG-PET).
Alternative diagnosis mimicking vasculitis should be excluded prior to applying criteria.

2.3 Supplementary Table 3. Radiologic criteria of abnormal wall thickness in arteritis

CT criteria	Positive if wall thickness	Ref
**Aorta**	> 2 mm without adjacent atherosclerotic plaque (or aortic aneurysm or ectasia)	(Agard et al., 2008)
	≥ 3 mm circumferential in absence of adjacent atherosclerotic plaque	(Marie et al., 2009)
	≥ 2 mm circumferential in areas without adjacent atheroma	(Prieto-Gonzalez et al., 2012)
**Aortic branches** (e.g. brachiocephalic trunk, carotids, subclavian, axillary arteries)	>1mm circumferential and contrast enhancement of artery wall	(Prieto-Gonzalez et al., 2012;Prieto-Gonzalez et al., 2015)

US criteria	Positive if intima-media thickness (IMT)	
**Common superficial temporal artery**	>0.42 mm (Se 100%; Sp 100%)	(Schafer et al., 2017)
**Frontal branch**	>0.34 mm (Se 100%; Sp 100%)	(Schafer et al., 2017)
**Parietal branch**	>0.29 mm (Se 97.2%; Sp 98.7%)	(Schafer et al., 2017)
**Facial artery**	>0.37 mm (Se 87.5%; Sp 98.8%)	(Schafer et al., 2017)
**Axillary artery**	>1.0 mm (Se 100%; Sp 100%)	(Schafer et al., 2017)

2.4 Supplementary Table 4. Stroke and relapse after steroid initiation in GCA cohorts.

GCA, n	GCA-associated stroke, % (n)	Relapses after steroids, % (n)	Criteria	Study setting	Ref.
—	(4)	(2)	Stroke	—	(Guisado-Alonso et al., 2021)
—	(25)	(11)	Stroke	Consec Multi (Germany, Swiss) - 2022	(Beuker et al., 2021)
N/A	(14)	(2)	Stroke	Database: Stroke and GCA. Multi (Paris, France) 2002-2016	(Chazal et al., 2018)
57	7.0 (4)[e]	1.8 (1)	Stroke	Consec Multi (Dijon, France) 2001-2011	(Samson et al., 2015)
70	4.2 (3)	N/A	Stroke	Consec Mono (Pekin, China) 1992-2014	(Sun et al., 2016)
97	8.2 (8)[c]	4.1 (4)	Stroke	Consec Mono (Paris Bichat, France) 2008-2014	(Lariviere et al., 2014)
98	5.1 (5) [a]	0 (0)	Stroke	Consec Mono (Valence, France) 1999-2012	(Zenone and Puget, 2013)
104	1.9 (2)	N/A	Stroke	Consec Mono (Basel, Switzerland) 1997-2007	(Berger et al., 2009)
166	2.4 (4)	0.6 (1)	Stroke	Consec Mono (Barcelona, Spain) 2012-2018	(Caselli et al., 1988)
175	7.4 (13)	3.4 (6)	Stroke	Consec Multi (Jerusalem, Israel) 1980-2000	(Nesher et al., 2004)
178	1.1 (2)	0.6 (1)	Stroke	Consec Mono (Limoges, France) 1976-2001	(Liozon et al., 2001)
180	2.8 (5)	N/A	Stroke, TIA	Consec Mono (Reggio Emilia, Italy) 1986-2005	(Salvarani et al., 2009)
200	1.5 (3)	(≥1?)	Stroke	Consec Mono (Barcelona, Spain) 1980-2016	(Cid et al., 1998)
244	4.1 (10)	10.2 (25)[d]	Stroke	Database (Olmsted, USA) 1950-2009	(Lo Gullo et al., 2016)
287	2.8 (8)	1.7 (5)	Stroke	Consec Mono (Lugo, Spain) 1981-2008	(Gonzalez-Gay et al., 2009)
1141	N/A	0.5 (6) [b]	Stroke (ICD9)	Insurance database, Ontario. 1995-2002	(Ray et al., 2005)
3408	3.9 (132)[f]	N/A	Stroke	Insurance database: GCA (UK) 1990-2010	(Tomasson et al., 2014)
254	5.9 (15)	4.7 (12)	Stroke	Consec Mono (Nantes, France) 1998-2018	(de Mornac et al., 2021)
129	13.9 (18)	8.5 (11)	Stroke, TIA	Consec Multi (Paris Tenon /S^{t}Antoine, France) 2010-2018	(Pariente et al., 2019)
291	3.1 (9)	N/A	Stroke	Consec. Mono (Ljubljana, Slovenia) 2011-2019	(Hocevar et al., 2020)
271	5.2 (14)	0.7 (2)	Stroke, TIA	Consec. Mono (Lille, France) 2010-2020	(Penet et al., 2023)

560	3.4 (19)	0.5 (3)	Stroke	Consec. Mono (Limoges, France) 1982-2021	(Parreau et al., 2022)

Adapted from (Soriano et al., 2017). **Abbreviations:** consec: consecutive cases; mono/multi: mono- or multicenter study; N/A: not assessed.

[a] one case excluded: occlusion of carotid artery, no stroke.

[b] 1.9 per 1000 person-years in GCA, compared with 0.9 per 1000 person-years in paired controls.

[c] three case excluded: atrial fibrillation (1); non-fulfilled ACR criteria (2).

[d] cumulative incidence 7.1±1.7% at 10 years follow-up.

[e] five excluded due to stroke before clinical onset of GCA (3) or >4 weeks after steroid initiation (2).

[f] 8.0 per 1000 person-years in GCA, compared with 6.3 per 1000 person-years in paired controls.

2.5 Supplementary Table 5. Atypical cases excluded from analysis.

	**Non-GCA granulomatosis**
**(Enzmann and Scott, 1977)**	51-year-old man with three months history of headache fever, dysphagia, and VI nerve palsy, then right stroke. ESR 42mm. CSF 103 WBC. Positive TAB. Arteriography: multiple intracranial stenosis and fusiform aneurysms. **[Proposed diagnosis: PACNS]**
**(Enzmann and Scott, 1977)**	60-year-old man. Fever, weight loss, headache, then became stuporous and died. ESR 97mm. CSF 25 WBC. Arteriography: multiple intracranial stenosis and fusiform aneurysms. Autopsy: intracranial giant-cell arteritis and innumerable small infarctions. **[Proposed diagnosis: PACNS]**
**(Chen et al., 2010)**	24-year-old man with severe headache and weakness. He had previous non-Hodgkin lymphoma. Multiple intracranial arteries were occluded. Autopsy revealed arteritis lesions with granulomas. **[Proposed diagnosis: PACNS or post-lymphoma granulomatous angiitis, see (Lopez-Chiriboga et al., 2018)]**
**(Yahyavi-Firouz-Abadi et al., 2009)**	39-year-old man with headache. Negative TAB and CSF. CRP 8 mg/L and ESR 106 mm/h. High resolution CT revealed beading and stenosis of ophthalmic, carotid and superficial temporal artery, which all resolved after steroids. **[Proposed diagnosis: other large arteries vasculitis, PACNS?]**
**(Browne et al., 2003)**	37-year-old man. Fever and headache for a few weeks. Infection by chlamydia pneumoniae treated by doxycycline. Acute onset with status epilepticus and massive right carotid infarction. ESR 21mm. No endocarditis. Autopsy: right carotid occlusion and granulomatous arteritis of right middle cerebral artery. Diffuse meningeal giant cell reaction and lymphocytic meningitis **[Proposed diagnosis: PACNS or VZV arteritis]**
**(Rondeau et al., 2001)**	64-year-old man with worsening cognitive signs over months, no general symptoms. CRP 66mg/L. Extensive white matter lesions involving temporal and occipital lobes on left side, with a minute involvement of the right side, and cortical preservation. After steroids, normalization was obtained without atrophy, definitively ruling out stroke diagnosis. Positive TAB. No brain biopsy, brain vessel imaging, or T2-FFE sequence. **[Proposed diagnosis: GCA and leukopathy from different mechanism]**
**(Wu et al., 2004)**	31-year-old woman with headache, hearing loss and TIA. Angiography demonstrated narrowing of cervical arteries and highly atypical dolichoectasia. Positive TAB. **[Proposed diagnosis: PACNS]**
**(Imakita et al., 1993)**	59-year-old man. Bentall's procedure due to aortic regurgitation and angor, complicated by spinal cord ischemia. Nine years later, left carotid stroke. Absence of biological inflammation (fibrinogen, CRP). Sudden death after abdominal pain. Autopsy: atherosclerosis, focal destruction of IEL, giant cells. Occlusion of carotid, middle and anterior cerebral arteries. Giant cells were observed in aorta sampled years before. **[atherosclerosis? other chronic cause of vasculitis?]**
	**False-positive halo sign**

**(Kishk et al., 2021)**	36-year-old female presenting with posterior stroke and halo sign on vertebral artery, albeit normal biological test, normal temporal artery and absence of general signs. **[Proposed diagnosis: vertebral dissection]**
	**Non-GCA related stroke**
**(Dziadkowiak et al., 2022)**	89-year-old woman suffering from permanent atrial fibrillation without anticoagulation developed a stroke by acute occlusion of left internal carotid and middle cerebral artery (M1/M2 occlusion) which were repermeabilized after thrombolysis/thrombectomy. Orbital masses, chronic occlusion of right internal carotid and pulmonary embolism were discovered. High CRP. TAB not done. GCA diagnosis uncertain. Probable cardiac embolic stroke due to procoagulant state. **[proposed diagnosis: AF embolus and possible GCA]**

Abbreviations: CSF: cerebrospinal fluid; ESR: erythrocyte sedimentation rate; IEL: internal elastic lamina; PACNS: primary angiitis of central nervous system; TAB: temporal artery biopsy; TIA: transient ischemic attack; WBC: white blood cells (per mm^3). One or more provisional retrospective diagnoses are proposed between square brackets.

3 Supplementary Figures

3.1 Supplementary Figure 1. Representative brain and cervical artery lesions in GCA

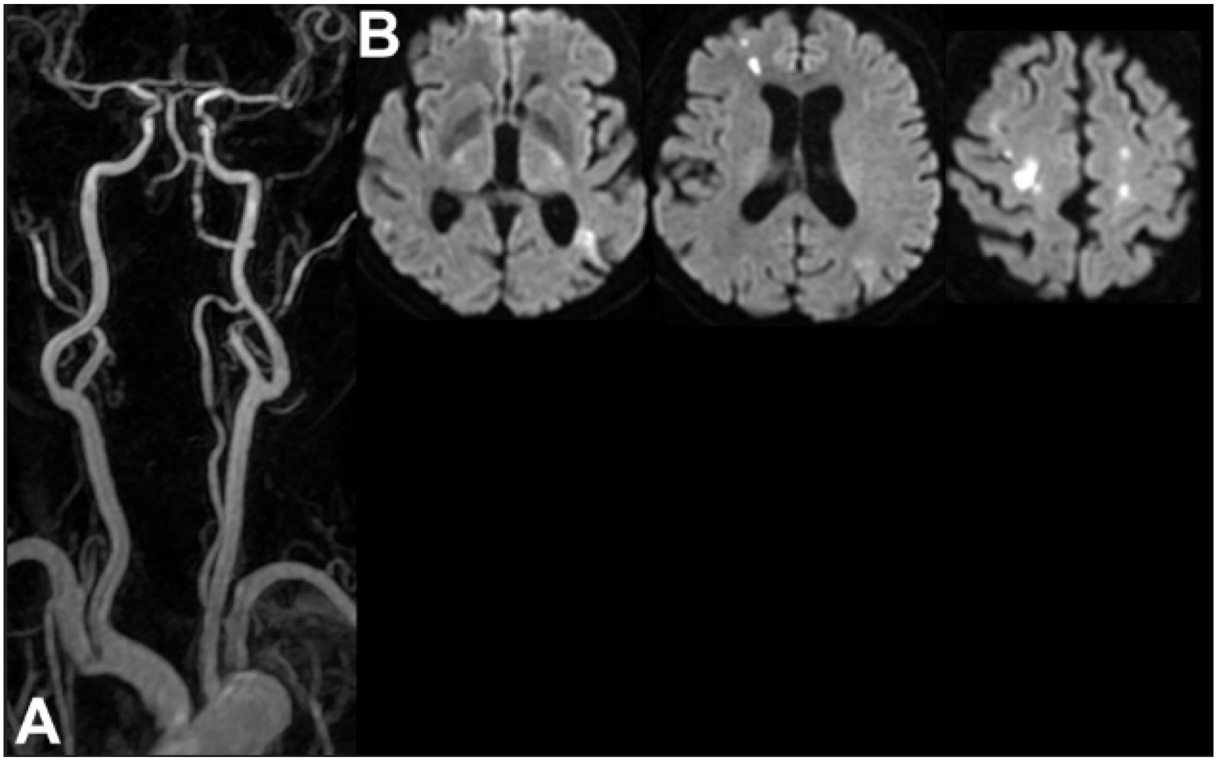

A. MR angiography. Occlusion of right vertebral artery (VA) up to distal V4 segment, irregular stenosis of left V4 and focal V2 segments, and bilateral stenosis of internal carotid siphons. **B.** Diffusion sequence. Bilateral acute ischemic lesions featuring a rosary-like pattern typical of watershed areas between MCA and ACA. (Patient #1).

3.2 Supplementary Figure 2. Illustrative case of GCA-related stroke.

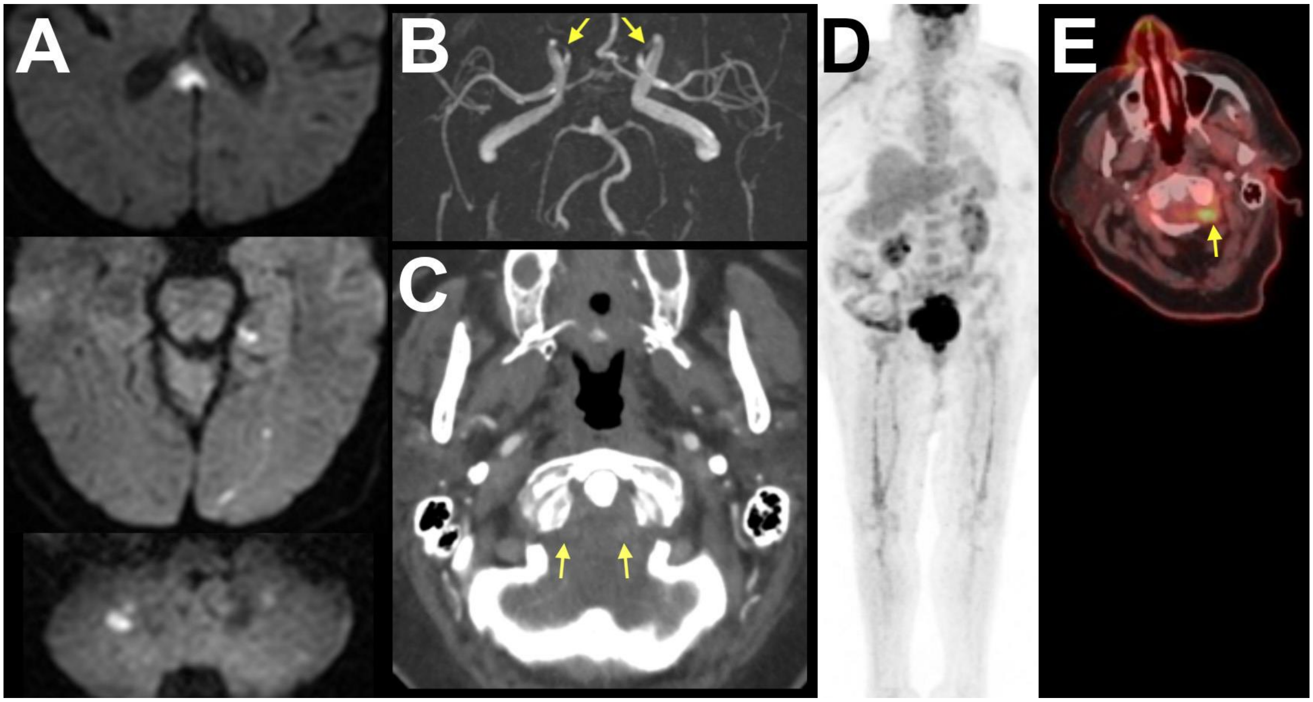

A woman on her seventies had tinnitus and minor headache for two weeks. She suddenly developed ataxic gait, minor aphasia, then became drowsy. Admitted at day 4. ESR 91mm/h, CRP 11mg/L (33mg/L at day 7). Positive TAB. **A.** MRI diffusion sequence. Bilateral very small acute ischemic lesions located in PICA and PCA territory. **B.** MR angiography. Bilateral stenosis of internal carotid siphons (arrows). **C.** Enhanced CT scan (arterial phase). Severe stenosis of vertebral arteries at the V3-V4 junction (arrows). **D.** Whole body FDG-PET. Vasculitis of femoral and vertebral arteries, and inflammation of soft tissues around the shoulders. **E.** FDG-PET/CT image. Uptake of left vertebral artery (V3 segment, arrow). (Patient #4).

3.3 Supplementary Figure 3. Representative CT and Pet-scans of GCA-associated strokes

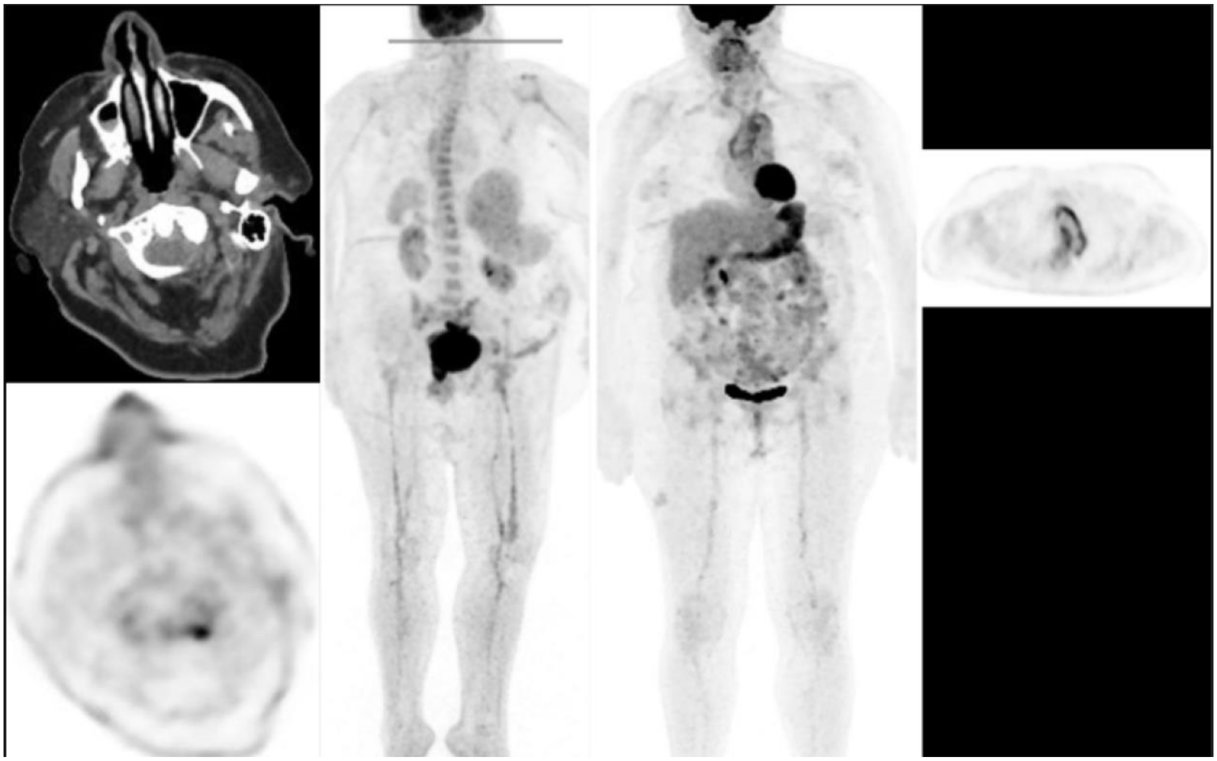

A-C. GCA mostly restricted to bilateral vertebral arteries: bilateral intense uptake of terminal V3 and proximal V4 segments, which are occluded. Asymmetric fixation is due to slightly oblique plane of acquisition. Minimal or absent uptake of aorta. (Patient #3).

D-E. GCA with major inflammation of aortic arch and cervical arteries (Patient #1).

4 References

Agard, C., Barrier, J.H., Dupas, B., Ponge, T., Mahr, A., Fradet, G., Chevalet, P., Masseau, A., Batard, E., Pottier, P., Planchon, B., Brisseau, J.M., and Hamidou, M.A. (2008). Aortic involvement in recent-onset giant cell (temporal) arteritis: a case-control prospective study using helical aortic computed tomodensitometric scan. *Arthritis Rheum* 59**,** 670-676.

Berger, C.T., Wolbers, M., Meyer, P., Daikeler, T., and Hess, C. (2009). High incidence of severe ischaemic complications in patients with giant cell arteritis irrespective of platelet count and size, and platelet inhibition. *Rheumatology (Oxford)* 48**,** 258-261.

Beuker, C., Wankner, M.C., Thomas, C., Strecker, J.K., Schmidt-Pogoda, A., Schwindt, W., Schulte-Mecklenbeck, A., Gross, C., Wiendl, H., Barth, P.J., Eckert, B., Meinel, T.R., Arnold, M., Schaumberg, J., Kruger, S., Deb-Chatterji, M., Magnus, T., Rother, J., and Minnerup, J. (2021). Characterization of Extracranial Giant Cell Arteritis with Intracranial Involvement and its Rapidly Progressive Subtype. *Ann Neurol* 90**,** 118-129.

Browne, L., Hardiman, O., O'dwyer, H., and Farrell, M. (2003). Intracranial giant cell arteritis with fatal middle cerebral artery territory infarct. *Clin Neuropathol* 22**,** 199-203.

Caselli, R.J., Hunder, G.G., and Whisnant, J.P. (1988). Neurologic disease in biopsy-proven giant cell (temporal) arteritis. *Neurology* 38**,** 352-359.

Chazal, T., Couture, P., Rosso, C., Haroche, J., Leger, A., Hervier, B., Deltour, S., Rufat, P., Amoura, Z., and Cohen Aubart, F. (2018). Cerebrovascular events are associated with lower survival in giant cell arteritis: A case-controlled multicenter study. *Joint Bone Spine* 85**,** 383-385.

Chen, Z.W., Symons, S.P., Young, B., and Bilbao, J.M. (2010). A 24-year-old male with headaches. *Brain Pathol* 20**,** 863-866.

Cid, M.C., Font, C., Oristrell, J., De La Sierra, A., Coll-Vinent, B., Lopez-Soto, A., Vilaseca, J., Urbano-Marquez, A., and Grau, J.M. (1998). Association between strong inflammatory response and low risk of developing visual loss and other cranial ischemic complications in giant cell (temporal) arteritis. *Arthritis Rheum* 41**,** 26-32.

De Mornac, D., Espitia, O., Neel, A., Connault, J., Masseau, A., Espitia-Thibault, A., Artifoni, M., Achille, A., Wahbi, A., Lacou, M., Durant, C., Pottier, P., Perrin, F., Graveleau, J., Hamidou, M., Hardouin, J.B., and Agard, C. (2021). Large-vessel involvement is predictive of multiple relapses in giant cell arteritis. *Ther Adv Musculoskelet Dis* 13**,** 1759720X211009029.

Dziadkowiak, E., Chojdak-Lukasiewicz, J., Paradowski, B., and Bladowska, J. (2022). Isolated arteritis misdiagnosed as bilateral orbital tumors in a patient with acute ischemic stroke. *Radiol Case Rep* 17**,** 3927-3932.

Enzmann, D., and Scott, W.R. (1977). Intracranial involvement of giant-cell arteritis. *Neurology* 27**,** 794-797.

Gonzalez-Gay, M.A., Vazquez-Rodriguez, T.R., Gomez-Acebo, I., Pego-Reigosa, R., Lopez-Diaz, M.J., Vazquez-Trinanes, M.C., Miranda-Filloy, J.A., Blanco, R., Dierssen, T., Gonzalez-Juanatey, C., and Llorca, J. (2009). Strokes at time of disease diagnosis in a series of 287 patients with biopsy-proven giant cell arteritis. *Medicine (Baltimore)* 88**,** 227-235.

Guisado-Alonso, D., Edo, M.C., Estrada Alarcon, P.V., Garcia-Sanchez, S.M., Font, M.A., Mena Romo, L., Marrero-Gonzalez, P., Mengual, J.J., Castrillo, L., Montull, C., Corominas, H., and Gomez-Choco, M. (2021). Progression of Large Vessel Disease in Patients With Giant Cell Arteritis-Associated Ischemic Stroke: The Role of Vascular Imaging: A Case Series. *J Clin Rheumatol* 27**,** e418-e424.

Hocevar, A., Jese, R., Tomsic, M., and Rotar, Z. (2020). Risk factors for severe cranial ischaemic complications in giant cell arteritis. *Rheumatology (Oxford)* 59**,** 2953-2959.

Hunder, G.G., Bloch, D.A., Michel, B.A., Stevens, M.B., Arend, W.P., Calabrese, L.H., Edworthy, S.M., Fauci, A.S., Leavitt, R.Y., Lie, J.T., and Et Al. (1990). The American College of

Rheumatology 1990 criteria for the classification of giant cell arteritis. *Arthritis Rheum* 33**,** 1122-1128.

Imakita, M., Yutani, C., and Ishibashi-Ueda, H. (1993). Giant cell arteritis involving the cerebral artery. *Arch Pathol Lab Med* 117**,** 729-733.

Kishk, N.A., Alsayyad, A., Alsayyad, E., and Hatem, G. (2021). Isolated vertebral arteritis: a challenging cause of stroke. *Acta Neurol Belg* 121**,** 283-286.

Lariviere, D., Sacre, K., Klein, I., Hyafil, F., Choudat, L., Chauveheid, M.P., and Papo, T. (2014). Extra- and intracranial cerebral vasculitis in giant cell arteritis: an observational study. *Medicine (Baltimore)* 93**,** e265.

Liozon, E., Herrmann, F.R., Ly, K., Jauberteau, M.O., Loustaud, V., Soria, P., Robert, P.Y., Liozon, F., and Vidal, E. (2001). [Risk factors for irreversible cerebral ischemia complications from Horton's disease: prospective study of 178 patients]. *Rev Med Interne* 22**,** 30-41.

Lo Gullo, A., Koster, M.J., Crowson, C.S., Makol, A., Ytterberg, S.R., Saitta, A., Salvarani, C., Matteson, E.L., and Warrington, K.J. (2016). Venous Thromboembolism and Cerebrovascular Events in Patients with Giant Cell Arteritis: A Population-Based Retrospective Cohort Study. *PLoS One* 11**,** e0149579.

Lopez-Chiriboga, A.S., Yoon, J.W., Siegel, J.L., Harriott, A.M., Pirris, S., Eidelman, B.H., and Freeman, W.D. (2018). Granulomatous Angiitis of the Central Nervous System Associated with Hodgkin's Lymphoma: Case Report and Literature Review. *J Stroke Cerebrovasc Dis* 27**,** e5-e8.

Marie, I., Proux, A., Duhaut, P., Primard, E., Lahaxe, L., Girszyn, N., Louvel, J.P., and Levesque, H. (2009). Long-term follow-up of aortic involvement in giant cell arteritis: a series of 48 patients. *Medicine (Baltimore)* 88**,** 182-192.

Nesher, G., Berkun, Y., Mates, M., Baras, M., Rubinow, A., and Sonnenblick, M. (2004). Low-dose aspirin and prevention of cranial ischemic complications in giant cell arteritis. *Arthritis Rheum* 50**,** 1332-1337.

Pariente, A., Guedon, A., Alamowitch, S., Thietart, S., Carrat, F., Delorme, S., Capron, J., Cacciatore, C., Soussan, M., Dellal, A., Fain, O., and Mekinian, A. (2019). Ischemic stroke in giant-cell arteritis: French retrospective study. *J Autoimmun* 99**,** 48-51.

Parreau, S., Dumonteil, S., Montoro, F.M., Gondran, G., Bezanahary, H., Palat, S., Ly, K.H., Fauchais, A.L., and Liozon, E. (2022). Giant cell arteritis-related stroke in a large inception cohort: A comparative study. *Semin Arthritis Rheum* 55**,** 152020.

Penet, T., Lambert, M., Baillet, C., Outteryck, O., Henon, H., Morell-Dubois, S., Hachulla, E., Launay, D., and Pokeerbux, M.R. (2023). Giant cell arteritis-related cerebrovascular ischemic events: a French retrospective study of 271 patients, systematic review of the literature and meta-analysis. *Arthritis Res Ther* 25**,** 116.

Ponte, C., Grayson, P.C., Robson, J.C., Suppiah, R., Gribbons, K.B., Judge, A., Craven, A., Khalid, S., Hutchings, A., Watts, R.A., Merkel, P.A., Luqmani, R.A., and Group, D.S. (2022). 2022 American College of Rheumatology/EULAR classification criteria for giant cell arteritis. *Ann Rheum Dis* 81**,** 1647-1653.

Prieto-Gonzalez, S., Arguis, P., Garcia-Martinez, A., Espigol-Frigole, G., Tavera-Bahillo, I., Butjosa, M., Sanchez, M., Hernandez-Rodriguez, J., Grau, J.M., and Cid, M.C. (2012). Large vessel

involvement in biopsy-proven giant cell arteritis: prospective study in 40 newly diagnosed patients using CT angiography. *Ann Rheum Dis* 71**,** 1170-1176.

Prieto-Gonzalez, S., Garcia-Martinez, A., Tavera-Bahillo, I., Hernandez-Rodriguez, J., Gutierrez-Chacoff, J., Alba, M.A., Murgia, G., Espigol-Frigole, G., Sanchez, M., Arguis, P., and Cid, M.C. (2015). Effect of glucocorticoid treatment on computed tomography angiography detected large-vessel inflammation in giant-cell arteritis. A prospective, longitudinal study. *Medicine (Baltimore)* 94**,** e486.

Ray, J.G., Mamdani, M.M., and Geerts, W.H. (2005). Giant cell arteritis and cardiovascular disease in older adults. *Heart* 91**,** 324-328.

Rondeau, M., Weber, J., and Storck, D. (2001). A case of giant cell arteritis associated with suspected cerebral angiitis. *Eur J Intern Med* 12**,** 459-461.

Salvarani, C., Della Bella, C., Cimino, L., Macchioni, P., Formisano, D., Bajocchi, G., Pipitone, N., Catanoso, M.G., Restuccia, G., Ghinoi, A., and Boiardi, L. (2009). Risk factors for severe cranial ischaemic events in an Italian population-based cohort of patients with giant cell arteritis. *Rheumatology (Oxford)* 48**,** 250-253.

Samson, M., Jacquin, A., Audia, S., Daubail, B., Devilliers, H., Petrella, T., Martin, L., Durier, J., Besancenot, J.F., Lorcerie, B., Giroud, M., Bonnotte, B., and Bejot, Y. (2015). Stroke associated with giant cell arteritis: a population-based study. *J Neurol Neurosurg Psychiatry* 86**,** 216-221.

Schafer, V.S., Juche, A., Ramiro, S., Krause, A., and Schmidt, W.A. (2017). Ultrasound cut-off values for intima-media thickness of temporal, facial and axillary arteries in giant cell arteritis. *Rheumatology (Oxford)* 56**,** 1632.

Soriano, A., Muratore, F., Pipitone, N., Boiardi, L., Cimino, L., and Salvarani, C. (2017). Visual loss and other cranial ischaemic complications in giant cell arteritis. *Nat Rev Rheumatol* 13**,** 476-484.

Sun, F., Ma, S., Zheng, W., Tian, X., and Zeng, X. (2016). A Retrospective Study of Chinese Patients With Giant Cell Arteritis (GCA): Clinical Features and Factors Associated With Severe Ischemic Manifestations. *Medicine (Baltimore)* 95**,** e3213.

Tomasson, G., Peloquin, C., Mohammad, A., Love, T.J., Zhang, Y., Choi, H.K., and Merkel, P.A. (2014). Risk for cardiovascular disease early and late after a diagnosis of giant-cell arteritis: a cohort study. *Ann Intern Med* 160**,** 73-80.

Wu, C.S., Hsu, K.L., Chang, Y.L., and Lee, K.L. (2004). Giant cell arteritis with CD8+ instead of CD4+ T lymphocytes as the predominant infiltrating cells in a young woman. *J Microbiol Immunol Infect* 37**,** 246-249.

Yahyavi-Firouz-Abadi, N., Wynn, B.L., Rybicki, F.J., Steigner, M.L., Hussain, A.Z., Mather, R., Hanson, E.H., Ansarinia, M., and Orrison, W.W., Jr. (2009). Steroid-responsive large vessel vasculitis: application of whole-brain 320-detector row dynamic volume CT angiography and perfusion. *AJNR Am J Neuroradiol* 30**,** 1409-1411.

Zenone, T., and Puget, M. (2013). Characteristics of cerebrovascular accidents at time of diagnosis in a series of 98 patients with giant cell arteritis. *Rheumatol Int* 33**,** 3017-3023.
