# Supplementary material for: Giant-cell arteritis related strokes: scoping review of mechanisms and rethinking treatment strategy?
Source: Front Neurol. 2023 Dec 7;14:1305093. doi: 10.3389/fneur.2023.1305093 (PMC10733536; doi:10.3389/fneur.2023.1305093)
Supplement: Supplementary file 2 [file Data_Sheet_2.pdf]

Supplementary Material 2

Giant-cell arteritis related strokes: scoping review of mechanisms and rethinking treatment strategy?

Mickael, BONNAN*, Stéphane, DEBEUGNY

*** Correspondence:** Corresponding Author: mickael_bonnan@yahoo.fr

1 Supplementary Data. Meta-analysis of risk factors for ischemic brain complications.

Influence of clinical and biological parameters upon the risk of brain ischemic events complicating GCA was examined. Meta-analyze faced to multiple limitations: most of series included a small number of patients in the group of ischemic events; brain strokes were often pooled with non-brain ischemic events (optic nerve or other crania nerves) or with transient optic or brain events; and all studies were descriptive whereas none of them was a randomized clinical trial (RCT).

1.1 Selection criteria

A systematic research of article dealing with brain ischemic events was made on Pubmed using the international standard of PRISMA (see PRISMA Flow Diagram in Supplementary Material 1). All series of GCA obtained from selected articles were included if two groups were distinguished based on ischemic events. Definition of *'ischemic events'* remained open and were highly variable according to the choice of transient or permanent events, ophthalmic events, or other ischemic events. Inclusion criteria of ischemic events were examined for each study (Supplementary Table 1). Median delay of onset of ischemic event from GCA onset or steroid initiation was highly heterogenous among studies.

Exclusion criteria are as follows: absence of stroke in the ischemic group, study size below 5 patients by group. Parameters available in less than two studies were excluded from analysis. Relevant data were extracted by one author (MB).

Quantitative data were: sample size, age at CGA onset, delay to diagnosis, platelet count, CRP, hemoglobin and ESR levels. When mean and/or standard deviation were unavailable, they were estimated using Hozo's method based on sample size, min, median and max values; or using Wan's method based on sample size, median and interquartile range (Wan et al., 2014).

Qualitative data were: transient signs (amaurosis fugax or TIA), abnormal temporal artery on physical examination, antiaggregation or anticoagulation before the diagnosis of GCA, aortitis, constitutional syndrome (asthenia, anorexia, weight loss>4kg), diabetes mellitus, fever (≥38°C), hypertension, smoking, hypercholesterolemia, headache, ischemic heart disease, jaw

claudication, ophthalmic signs, irreversible visual loss, polymyalgia rheumatica, positive temporal artery biopsy (TAB), scalp tenderness.

1.2 Estimation of biases

Since included studies were based on consecutive GCA patients split by ischemic events and since most the data were common clinical and biological parameters, bias in the selection of patient participant to the study, bias due to missing data and bias in selection of the reported patient were low. Bias in the measurement of outcome may have occurred in ill or subjectively defined clinical signs, like scalp tenderness or jaw claudication.

Bias due to confounding effect may occurred for transient ischemic signs, which were clinical grounded, and may have influenced the risk of persisting ischemic event also defined by radiology.

Definition of ischemic event was variable among studies especially by the variable inclusion, besides strokes, of ophthalmic or other cranial ischemic events, potentially generating a bias in classification of patients. Admitting reasonably similar mechanisms of ischemic events, the direction of the bias is likely to increase the effect estimate.

1.3 Statistical analysis

The estimated pooled effect of each parameter is calculated using the mean of standardized effect of each study weighted by the inverse of variance. Statistic heterogeneity is evaluated by the Cochran's Q test, which is complemented with the I^2 statistic: heterogeneity values of I^2 of 25%, 50%, and 75% was designated as low, moderate and high. Assuming a high level of heterogeneity, random effect model was used to assess the pooled effect and 95% confidence interval (CI). The presence of publication bias was evaluated by funnel plots. For each risk factor, results were given as forest plots. *P*-value below 0.05 was considered significant. All statistical analyses are performed by SAS software version 9.4 (SAS Institute, Cary, NC, USA) and Review Manager (RevMan) 5.4.1 software from the Cochrane Collaboration. Results are given in odds ratio, 95% confidence interval (OR [95% CI]).

1.4 Results

Twenty-two studies published between 1991 and 2023 were extracted from the literature, and one was excluded due to absence of stroke from cranial ischemic events.

Forest plots and odds ratio for risk factors of ischemic events associated GCA are given below in Suppl. Table S2 and Forest plots (for discussion: see Main document).

2 Supplementary Tables

2.1.1 Supplementary Table S1. Inclusion criteria of ischemic events in selected studies.

Authors	Amo. Fug.	Irreversible ophthalmic	TIA	Stroke	Cranial nerves	Other ischemic signs	Clear definition	Other criteria	Delay criteria
(Berger et al., 2009)	yes	yes	yes	yes	n/s	JC	yes	—	'occurring within two weeks of diagnosis'
(Chazal et al., 2018)	n/s	n/s	n/s	yes (?)	n/s	—	no	Cerebrovascular event, no AF or atheroma mechanism	n/s
(Cid et al., 1998)	yes	yes	yes	yes	yes	heart, limb, scalp	yes	absence of obvious vascular risk factors	'concomitant’
(de Boysson et al., 2017)	no	no	no	yes	n/s	—	yes	—	GCA onset to week 4 post-CS initiation
(De Keyser et al., 1991)	yes	yes	yes	yes	no	—	yes	—	n/s
(de Mornac et al., 2021)	n/s	n/s	yes	yes	n/s	aortic, heart, limb, digestive	yes	—	n/s
(Gonzalez-Gay et al., 1998)	yes	yes	yes	yes	yes	—	yes	—	n/s

(Gonzalez-Gay et al., 2009)	no	no	no	yes	yes	—	yes	—	GCA onset to week 4 post-CS initiation
(Hayreh et al., 1998)	yes	yes	no	no	yes	—	no	—	n/s
(Hocevar et al., 2020)	yes	yes	yes	yes	yes	no	yes	—	
(Lee et al., 2006)	yes	yes	no	yes	yes	—	yes	—	'if signs, symptoms, or laboratory evidence of a recurrence was present'
(Liozon et al., 2001)	no	yes	no	yes	yes	deafness	yes	—	n/s
(Nesher and Sonnenblick, 1998)	no	yes	no	yes	n/s	tongue	yes	—	n/s
(Nesher et al., 2004)	yes	yes	yes	yes	yes	—	yes	—	GCA onset to week 2 post-CS initiation, or with ≥1 sign of GCA relapse
(Pariente et al., 2019)	no	no	yes	yes	n/s	—	yes	no AF	GCA onset to month 12 post-CS initiation

(Parreau et al., 2022)	no	no	no	yes	no	—	yes	no AF	≤1month prior GCA onset and within ≤4weeks post-CS initiation
(Pego-Reigosa et al., 2004)	no	no	yes	yes	n/s	—	yes	—	n/s
(Penet et al., 2023)	no	no	no	yes	no	—	yes	—	GCA onset to week 4 post-CS initiation
(Salvarani et al., 2009)	yes	yes	yes	yes	n/s	—	yes	—	GCA onset to week 4 post-CS initiation
(Sun et al., 2016)	no	yes	no	yes	n/s	JC	yes	—	n/s
(Zenone and Puget, 2013)	no	no	yes	yes	n/s	—	yes	—	n/s

Abbreviations: AF: atrial fibrillation; Amo.Fug.: amaurosis fugax; JC: jaw claudication; n/s: not stated; TIA: transient ischemic attack.

2.1.2 Supplementary Table S2. Risk factors of ischemic events associated GCA.

Conditions	Heterogeneity, I^2 (%)	*P* [a]	Odds ratio, [95% CI]	*p*
Transient signs	0	0.52	5.03 [2.81, 9.01]	$<10^{-5}$
Chronic hypertension	0	0.70	1.82 [1.38, 2.41]	$<10^{-4}$
Visual loss	0	0.42	2.38 [1.41, 4.01]	0.001
Polymyalgia rheumatica	0	0.59	0.66 [0.51, 0.85]	0.001
Constitutional syndrome	38	0.09	0.64 [0.47, 0.88]	0.005
Aortitis	0	0.67	3.09 [1.39, 6.85]	0.005
Diabetes mellitus	0	0.63	1.65 [1.15, 2.36]	0.006
Positive TAB	18	0.28	1.69 [1.11, 2.56]	0.01
Fever	48	0.03	0.60 [0.40, 0.91]	0.01
Atrial fibrillation	0	0.65	2.23 [1.20, 4.13]	0.01
Smoking	33	0.18	1.69 [1.06, 2.68]	0.03
Hypercholesterolemia	0	0.53	1.37 [1.02, 1.84]	0.04
Scalp tenderness	0	0.64	0.70 [0.50, 1.00]	0.05
Ophthalmic signs	71	0.02	1.88 [0.77, 4.60]	0.17
Headache	54	0.007	0.78 [0.54, 1.12]	0.17
Jaw claudication	49	0.02	1.19 [0.87, 1.64]	0.27
Abnormal temporal artery on physical examination	28	0.18	1.27 [0.88, 1.82]	0.20
Antiaggreg./coag.[b]	66	0.007	1.32 [0.73, 2.41]	0.36
Ischemic heart disease	29	0.21	1.30 [0.67, 2.53]	0.43

Conditions	Heterogeneity, I^2 (%)	*P* [a]	Mean difference, [95% CI]	*p*
CRP (mg/L)	73	<0.001	-29.01 [-42.61, -15.41]	<0.001
Hemoglobin (g/dL)	64	0.001	0.49 [0.19, 0.79]	0.001
ESR (mm/h)	57	0.002	-5.70 [-9.71, -1.69]	0.005
Age at diagnosis (years)	59	0.001	1.48 [0.36, 2.60]	0.01
Delay to diagnosis (weeks)	0	0.77	-1.17 [-3.68, 1.34]	0.36
Albumin (g/L)	78	0.03	0.19 [-0.44, 0.81]	0.56
Platelets (G/L)	78	<0.001	1.48 [-44.33, 47.28]	0.95

[a] Cochrane Q test. [b] antiaggregation or anticoagulation before the diagnosis.

3 Forest plots

3.1 Figure S1. Forest plot showing the risk of ischemic complication associated with chronic hypertension.

Study or Subgroup	with ischemic event Events	with ischemic event Total	without ischemic event Events	without ischemic event Total	Weight	Odds Ratio M-H, Random, 95% CI
Chazal 2018	10	14	8	14	3.2%	1.88 [0.39, 9.01]
DeBoysson 2017	27	40	115	198	15.0%	1.50 [0.73, 3.08]
DeMornac 2021	23	39	85	215	16.1%	2.20 [1.10, 4.40]
Gonzalez-Gay 2009	5	8	107	279	3.7%	2.68 [0.63, 11.44]
Hocevar 2020	39	61	120	234	22.9%	1.68 [0.94, 3.01]
Pariente 2019	10	18	39	111	7.6%	2.31 [0.84, 6.32]
Parreau 2022	11	19	250	541	9.1%	1.60 [0.63, 4.04]
Penet 2023	6	14	145	271	6.6%	0.65 [0.22, 1.93]
Salvarani 2009	27	32	78	129	7.5%	3.53 [1.28, 9.77]
Sun 2016	16	29	15	41	8.3%	2.13 [0.81, 5.62]
**Total (95% CI)**		**274**		**2033**	**100.0%**	**1.82 [1.38, 2.41]**
Total events	174		962			

Heterogeneity: Tau² = 0.00; Chi² = 6.37, df = 9 (P = 0.70); I² = 0%
Test for overall effect: Z = 4.21 (P < 0.0001)

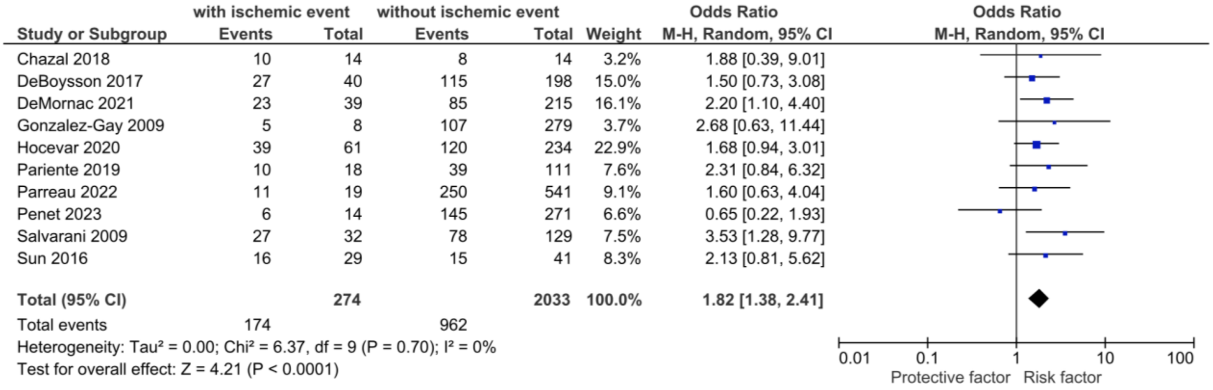

3.2 Figure S2. Forest plot showing the risk of ischemic complication associated with transient signs.

Study or Subgroup	with ischemic events Events	with ischemic events Total	without ischemic events Events	without ischemic events Total	Weight	Odds Ratio M-H, Random, 95% CI
Cid 1998	10	32	10	168	35.0%	7.18 [2.69, 19.20]
Gonzalez-Gay 2009	2	8	34	279	12.6%	2.40 [0.47, 12.38]
Nesher 2004	17	43	16	132	52.4%	4.74 [2.12, 10.60]
**Total (95% CI)**		**83**		**579**	**100.0%**	**5.03 [2.81, 9.01]**
Total events	29		60			

Heterogeneity: Tau² = 0.00; Chi² = 1.31, df = 2 (P = 0.52); I² = 0%
Test for overall effect: Z = 5.44 (P < 0.00001)

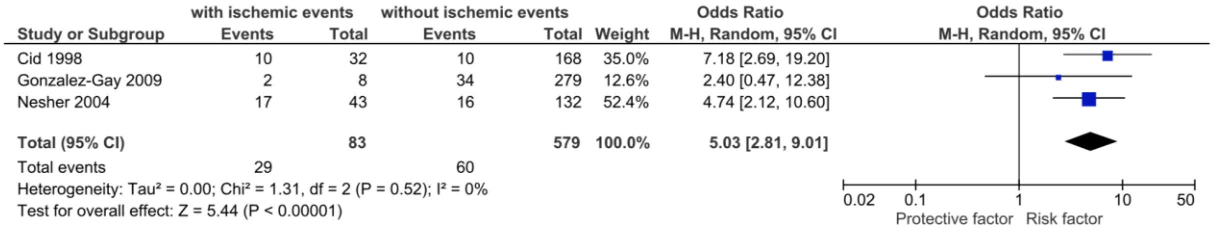

3.3 Figure S3. Forest plot showing the risk of ischemic complication associated with visual loss.

Study or Subgroup	with ischemic event Events	with ischemic event Total	without ischemic event Events	without ischemic event Total	Weight	Odds Ratio M-H, Random, 95% CI
Gonzalez-Gay 2009	3	8	33	279	12.5%	4.47 [1.02, 19.58]
Pariente 2019	6	18	12	111	20.7%	4.13 [1.31, 13.01]
Parreau 2022	6	19	83	541	27.6%	2.55 [0.94, 6.89]
Pego-Reigosa 2004	6	30	21	180	27.1%	1.89 [0.69, 5.16]
Penet 2023	1	14	40	271	6.4%	0.44 [0.06, 3.49]
Zenone 2013	1	6	14	92	5.5%	1.11 [0.12, 10.27]
**Total (95% CI)**		**95**		**1474**	**100.0%**	**2.38 [1.41, 4.01]**
Total events	23		203			

Heterogeneity: Tau² = 0.00; Chi² = 4.97, df = 5 (P = 0.42); I² = 0%
Test for overall effect: Z = 3.25 (P = 0.001)

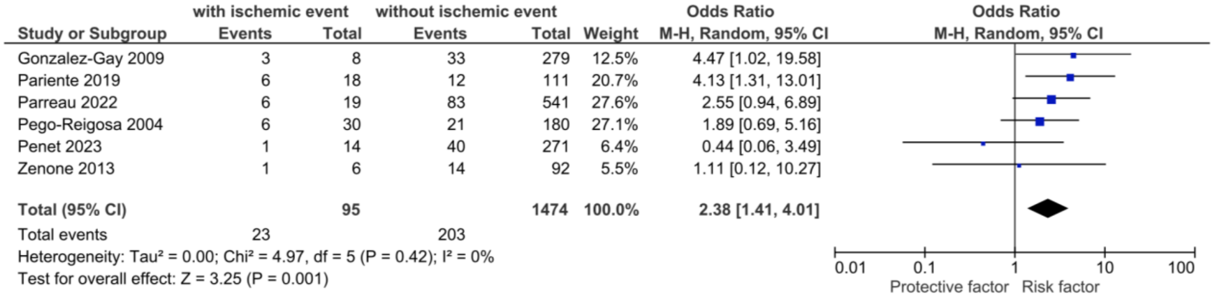

3.4 Figure S4. Forest plot showing the risk of ischemic complication associated with constitutional syndrome.

	with ischemic event		without ischemic event			Odds Ratio
Study or Subgroup	Events	Total	Events	Total	Weight	M-H, Random, 95% CI
DeMornac 2021	15	39	100	212	10.7%	0.70 [0.35, 1.41]
Gonzalez-Gay 1998	4	8	168	279	4.0%	0.66 [0.16, 2.70]
Gonzalez-Gay 2009	29	85	40	85	12.1%	0.58 [0.31, 1.08]
Hayreh 1998	3	18	21	83	4.4%	0.59 [0.16, 2.24]
Hocevar 2020	37	61	178	234	12.6%	0.49 [0.27, 0.88]
Nesher 1998	20	38	110	142	9.9%	0.32 [0.15, 0.68]
Nesher 2004	15	43	83	132	10.3%	0.32 [0.15, 0.65]
Parreau 2022	15	19	392	541	5.8%	1.43 [0.47, 4.36]
Pego-Reigosa 2004	3	6	23	92	3.0%	3.00 [0.57, 15.91]
Penet 2023	9	14	141	271	5.8%	1.66 [0.54, 5.08]
Salvarani 2009	42	68	126	170	12.5%	0.56 [0.31, 1.03]
Zenone 2013	20	30	115	180	8.9%	1.13 [0.50, 2.56]
**Total (95% CI)**		**429**		**2421**	**100.0%**	**0.64 [0.47, 0.88]**
Total events	212		1497			

Heterogeneity: Tau² = 0.11; Chi² = 17.77, df = 11 (P = 0.09); I² = 38%
Test for overall effect: Z = 2.80 (P = 0.005)

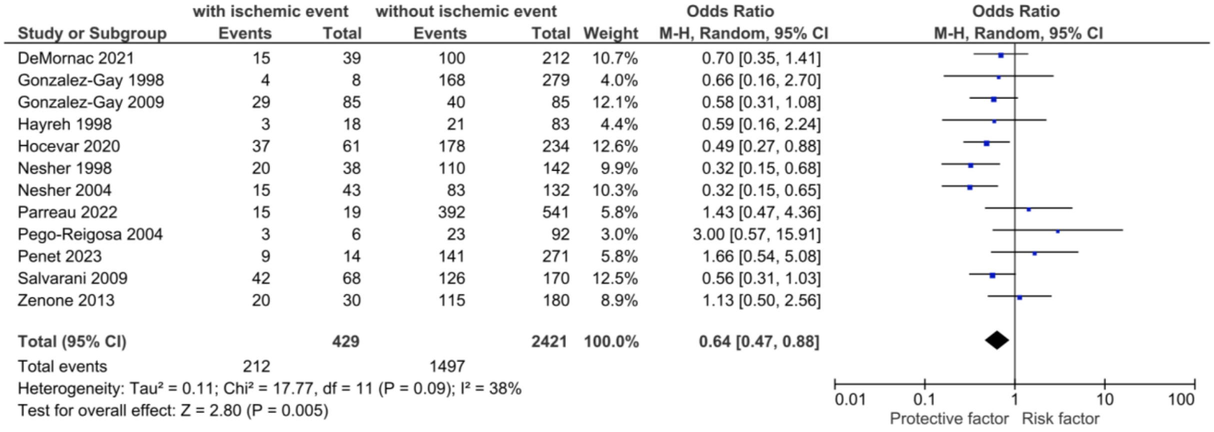

3.5 Figure S5. Forest plot showing the risk of ischemic complication associated with diabetes mellitus.

	with ischemic event		without ischemic event			Odds Ratio
Study or Subgroup	Events	Total	Events	Total	Weight	M-H, Random, 95% CI
Chazal 2018	3	14	4	14	4.3%	0.68 [0.12, 3.83]
DeBoysson 2017	8	40	26	198	16.7%	1.65 [0.69, 3.98]
DeMornac 2021	9	39	19	215	16.5%	3.09 [1.28, 7.47]
Gonzalez-Gay 2009	1	8	24	279	2.8%	1.52 [0.18, 12.86]
Hocevar 2020	7	61	29	234	16.7%	0.92 [0.38, 2.21]
Pariente 2019	5	18	15	111	9.4%	2.46 [0.77, 7.90]
Parreau 2022	5	19	61	541	11.5%	2.81 [0.98, 8.07]
Penet 2023	3	14	51	271	7.5%	1.18 [0.32, 4.37]
Salvarani 2009	3	37	7	136	6.5%	1.63 [0.40, 6.62]
Sun 2016	5	29	7	41	8.1%	1.01 [0.29, 3.57]
**Total (95% CI)**		**279**		**2040**	**100.0%**	**1.65 [1.15, 2.36]**
Total events	49		243			

Heterogeneity: Tau² = 0.00; Chi² = 7.03, df = 9 (P = 0.63); I² = 0%
Test for overall effect: Z = 2.74 (P = 0.006)

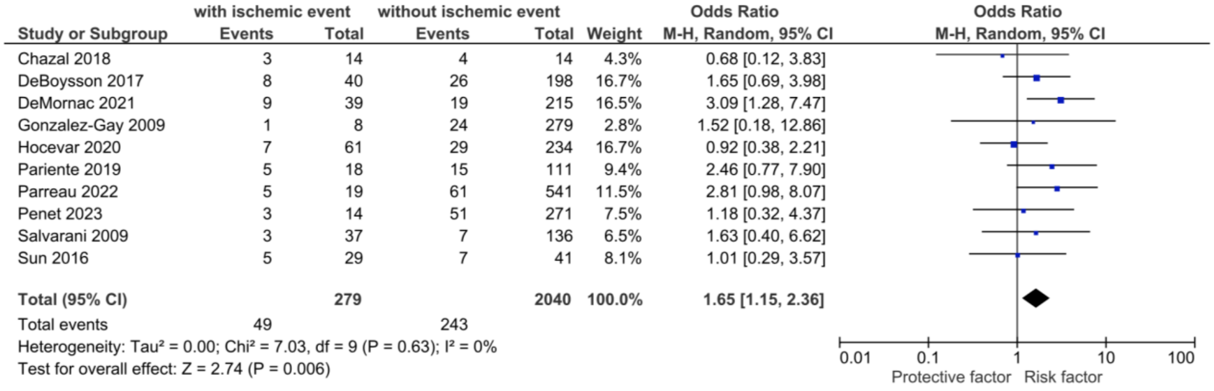

3.6 Figure S6. Forest plot showing the risk of ischemic complication associated with positive TAB.

	with ischemic event		without ischemic event			Odds Ratio
Study or Subgroup	Events	Total	Events	Total	Weight	M-H, Random, 95% CI
Chazal 2018	11	14	7	14	5.8%	3.67 [0.70, 19.12]
DeBoysson 2017	33	40	134	200	16.8%	2.32 [0.98, 5.53]
DeMornac 2021	27	38	140	210	20.3%	1.23 [0.58, 2.62]
Hocevar 2020	38	40	107	131	6.9%	4.26 [0.96, 18.89]
Nesher 2004	35	43	117	132	15.0%	0.56 [0.22, 1.43]
Pariente 2019	14	18	66	111	10.5%	2.39 [0.74, 7.72]
Parreau 2022	16	19	368	541	9.5%	2.51 [0.72, 8.72]
Penet 2023	8	14	129	271	11.9%	1.47 [0.50, 4.34]
Zenone 2013	5	6	63	92	3.4%	2.30 [0.26, 20.60]
**Total (95% CI)**		**232**		**1702**	**100.0%**	**1.69 [1.11, 2.56]**
Total events	187		1131			

Heterogeneity: Tau² = 0.07; Chi² = 9.77, df = 8 (P = 0.28); I² = 18%
Test for overall effect: Z = 2.47 (P = 0.01)

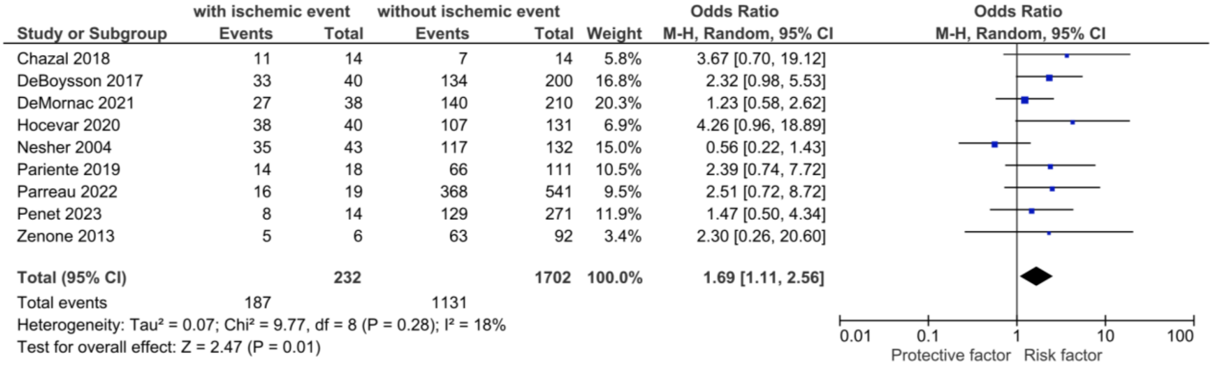

3.7 Figure S7. Forest plot showing the risk of ischemic complication associated with fever.

Study or Subgroup	with ischemic events Events	with ischemic events Total	without ischemic events Events	without ischemic events Total	Weight	Odds Ratio M-H, Random, 95% CI
Chazal 2018	4	14	8	14	4.9%	0.30 [0.06, 1.44]
Cid 1998	6	32	96	168	9.1%	0.17 [0.07, 0.44]
DeBoysson 2017	10	40	53	200	10.6%	0.92 [0.42, 2.02]
DeMornac 2021	14	38	68	214	11.3%	1.25 [0.61, 2.57]
Gonzalez-Gay 2009	0	8	32	279	1.8%	0.45 [0.03, 7.94]
Hayreh 1998	12	85	26	85	10.8%	0.37 [0.17, 0.80]
Hocevar 2020	9	61	60	234	10.8%	0.50 [0.23, 1.08]
Nesher 1998	3	18	23	83	6.1%	0.52 [0.14, 1.97]
Parreau 2022	8	19	192	541	9.2%	1.32 [0.52, 3.34]
Pego-Reigosa 2004	4	30	17	180	7.2%	1.48 [0.46, 4.73]
Penet 2023	2	14	51	271	5.1%	0.72 [0.16, 3.31]
Sun 2016	11	29	29	41	8.5%	0.25 [0.09, 0.69]
Zenone 2013	3	6	42	92	4.5%	1.19 [0.23, 6.21]
**Total (95% CI)**		**394**		**2402**	**100.0%**	**0.60 [0.40, 0.91]**
Total events	86		697			

Heterogeneity: Tau² = 0.25; Chi² = 23.13, df = 12 (P = 0.03); I² = 48%
Test for overall effect: Z = 2.43 (P = 0.01)

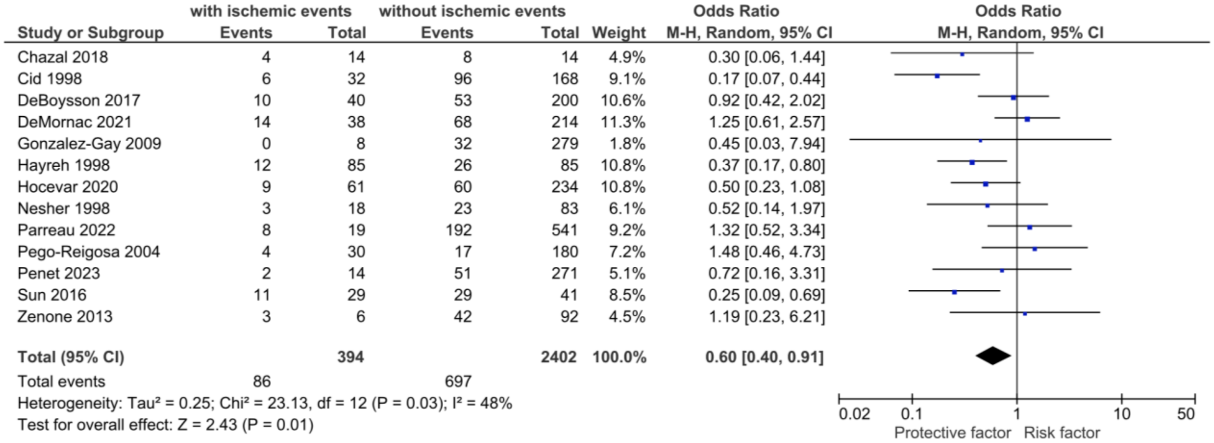

3.8 Figure S8. Forest plot showing the risk of ischemic complication associated with aortitis.

Study or Subgroup	with ischemic event Events	with ischemic event Total	without ischemic event Events	without ischemic event Total	Weight	Odds Ratio M-H, Random, 95% CI
Pariente 2019	3	18	7	111	29.9%	2.97 [0.69, 12.75]
Parreau 2022	6	19	61	541	63.0%	3.63 [1.33, 9.91]
Zenone 2013	0	6	7	92	7.2%	0.88 [0.04, 17.12]
**Total (95% CI)**		**43**		**744**	**100.0%**	**3.09 [1.39, 6.85]**
Total events	9		75			

Heterogeneity: Tau² = 0.00; Chi² = 0.81, df = 2 (P = 0.67); I² = 0%
Test for overall effect: Z = 2.78 (P = 0.005)

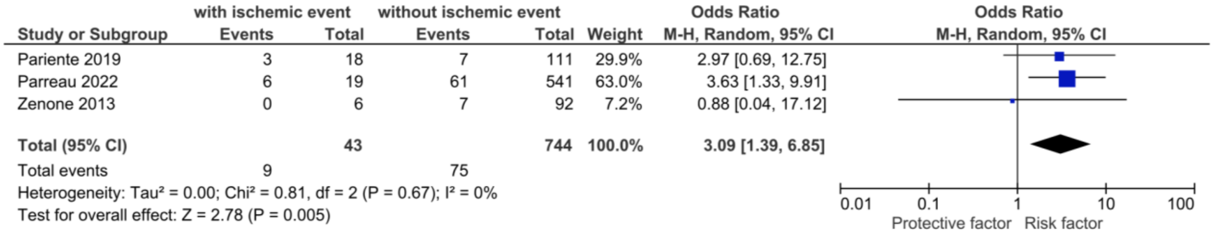

3.9 Figure S9. Forest plot showing the risk of ischemic complication associated with polymyalgia rheumatica.

Study or Subgroup	with ischemic event Events	with ischemic event Total	without ischemic event Events	without ischemic event Total	Weight	Odds Ratio M-H, Random, 95% CI
Cid 1998	15	32	83	168	11.2%	0.90 [0.42, 1.93]
DeBoysson 2017	8	40	76	198	9.4%	0.40 [0.18, 0.92]
DeMornac 2021	7	38	49	213	8.3%	0.76 [0.31, 1.82]
Gonzalez-Gay 1998	26	69	87	170	19.7%	0.58 [0.33, 1.02]
Gonzalez-Gay 2009	5	8	109	279	3.1%	2.60 [0.61, 11.10]
Hocevar 2020	6	61	40	234	7.8%	0.53 [0.21, 1.31]
Nesher 2004	10	43	53	132	10.4%	0.45 [0.21, 0.99]
Pariente 2019	4	18	19	111	4.4%	1.38 [0.41, 4.67]
Parreau 2022	3	19	172	541	4.1%	0.40 [0.12, 1.40]
Pego-Reigosa 2004	9	30	78	180	9.3%	0.56 [0.24, 1.29]
Penet 2023	4	14	89	271	4.6%	0.82 [0.25, 2.68]
Sun 2016	8	29	11	41	5.7%	1.04 [0.36, 3.02]
Zenone 2013	2	6	28	92	2.1%	1.14 [0.20, 6.61]
**Total (95% CI)**		**407**		**2630**	**100.0%**	**0.66 [0.51, 0.85]**
Total events	107		894			

Heterogeneity: Tau² = 0.00; Chi² = 10.29, df = 12 (P = 0.59); I² = 0%
Test for overall effect: Z = 3.19 (P = 0.001)

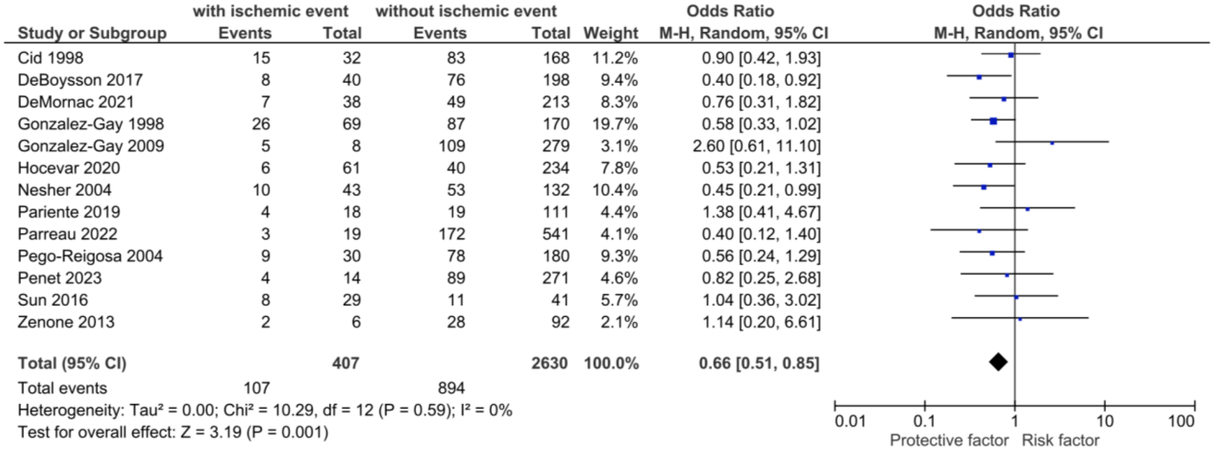

3.10 Figure S10 Forest plot showing the risk of ischemic complication associated with smoking.

Study or Subgroup	with ischemic event Events	with ischemic event Total	without ischemic event Events	without ischemic event Total	Weight	Odds Ratio M-H, Random, 95% CI
DeBoysson 2017	13	40	42	188	20.6%	1.67 [0.79, 3.53]
DeMornac 2021	5	39	32	215	14.2%	0.84 [0.31, 2.31]
Gonzalez-Gay 2009	4	8	41	279	8.5%	5.80 [1.40, 24.14]
Pariente 2019	7	18	33	111	13.8%	1.50 [0.54, 4.22]
Penet 2023	7	14	76	271	12.9%	2.57 [0.87, 7.56]
Salvarani 2009	7	37	30	134	16.1%	0.81 [0.32, 2.02]
Sun 2016	14	29	10	41	14.0%	2.89 [1.04, 8.02]
**Total (95% CI)**		**185**		**1239**	**100.0%**	**1.69 [1.06, 2.68]**
Total events	57		264			

Heterogeneity: Tau² = 0.12; Chi² = 8.90, df = 6 (P = 0.18); I² = 33%
Test for overall effect: Z = 2.22 (P = 0.03)

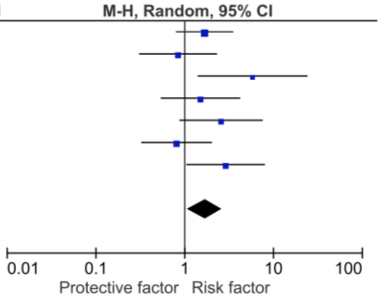

3.11 Figure S11. Forest plot showing the risk of ischemic complication associated with hypercholesterolemia.

Study or Subgroup	with ischemic event Events	with ischemic event Total	without ischemic event Events	without ischemic event Total	Weight	Odds Ratio M-H, Random, 95% CI
Chazal 2018	6	14	4	14	3.5%	1.88 [0.39, 9.01]
DeBoysson 2017	12	40	51	198	15.6%	1.24 [0.58, 2.61]
DeMornac 2021	13	39	46	215	15.8%	1.84 [0.88, 3.86]
Gonzalez-Gay 2009	3	8	46	279	4.1%	3.04 [0.70, 13.16]
Hocevar 2020	19	61	70	234	23.4%	1.06 [0.58, 1.95]
Pariente 2019	5	18	19	111	6.7%	1.86 [0.59, 5.84]
Parreau 2022	5	19	52	541	7.7%	3.36 [1.16, 9.70]
Penet 2023	5	14	94	271	6.9%	1.05 [0.34, 3.21]
Salvarani 2009	4	38	19	134	6.7%	0.71 [0.23, 2.24]
Sun 2016	14	29	21	41	9.6%	0.89 [0.34, 2.30]
**Total (95% CI)**		**280**		**2038**	**100.0%**	**1.37 [1.02, 1.84]**
Total events	86		422			

Heterogeneity: Tau² = 0.00; Chi² = 8.00, df = 9 (P = 0.53); I² = 0%
Test for overall effect: Z = 2.09 (P = 0.04)

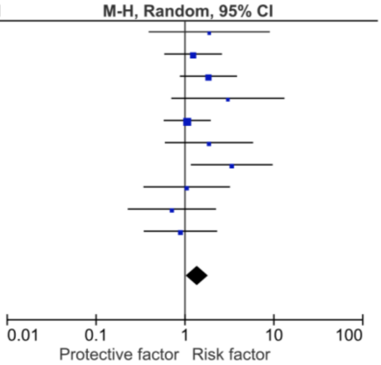

3.12 Figure S12. Forest plot showing the risk of ischemic complication associated with antiaggregation or anticoagulation before the diagnosis.

Study or Subgroup	with ischemic events Events	with ischemic events Total	without ischemic events Events	without ischemic events Total	Weight	Odds Ratio M-H, Random, 95% CI
DeBoysson 2017	13	40	72	200	17.4%	0.86 [0.42, 1.76]
DeMornac 2021	23	38	121	209	17.6%	1.12 [0.55, 2.26]
Gonzalez-Gay 2009	1	8	28	279	5.9%	1.28 [0.15, 10.79]
Hocevar 2020	16	61	41	234	18.2%	1.67 [0.86, 3.25]
Nesher 2004	3	43	33	132	11.7%	0.23 [0.07, 0.78]
Pariente 2019	9	18	22	111	13.8%	4.05 [1.44, 11.39]
Salvarani 2009	10	37	16	140	15.4%	2.87 [1.18, 7.01]
**Total (95% CI)**		**245**		**1305**	**100.0%**	**1.32 [0.73, 2.41]**
Total events	75		333			

Heterogeneity: Tau² = 0.40; Chi² = 17.71, df = 6 (P = 0.007); I² = 66%
Test for overall effect: Z = 0.91 (P = 0.36)

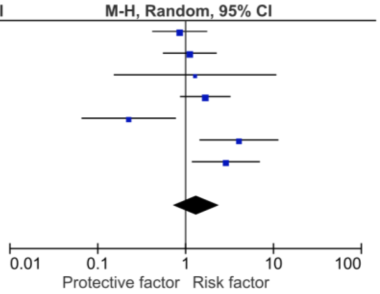

3.13 Figure S13. Forest plot showing the risk of ischemic complication associated with ophthalmic signs.

Study or Subgroup	with ischemic event Events	with ischemic event Total	without ischemic event Events	without ischemic event Total	Weight	Odds Ratio M-H, Random, 95% CI
Chazal 2018	6	14	4	14	17.2%	1.88 [0.39, 9.01]
DeBoysson 2017	25	40	50	200	29.5%	5.00 [2.44, 10.23]
DeMornac 2021	6	39	40	214	26.0%	0.79 [0.31, 2.02]
Pego-Reigosa 2004	9	30	40	180	27.3%	1.50 [0.64, 3.53]
**Total (95% CI)**		**123**		**608**	**100.0%**	**1.88 [0.77, 4.60]**
Total events	46		134			

Heterogeneity: Tau² = 0.57; Chi² = 10.46, df = 3 (P = 0.02); I² = 71%
Test for overall effect: Z = 1.39 (P = 0.17)

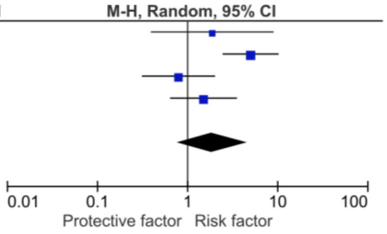

3.14 Figure S14. Forest plot showing the risk of ischemic complication associated with ischemic heart disease.

Study or Subgroup	with ischemic event Events	with ischemic event Total	without ischemic event Events	without ischemic event Total	Weight	Odds Ratio M-H, Random, 95% CI
Chazal 2018	3	14	2	14	9.3%	1.64 [0.23, 11.70]
DeBoysson 2017	6	40	22	198	24.5%	1.41 [0.53, 3.74]
Hocevar 2020	3	61	17	234	18.0%	0.66 [0.19, 2.33]
Parreau 2022	1	19	50	541	8.8%	0.55 [0.07, 4.17]
Penet 2023	0	14	11	271	4.8%	0.78 [0.04, 13.92]
Salvarani 2009	7	36	6	137	20.0%	5.27 [1.65, 16.85]
Sun 2016	3	29	6	41	14.5%	0.67 [0.15, 2.94]
**Total (95% CI)**		**213**		**1436**	**100.0%**	**1.30 [0.67, 2.53]**
Total events	23		114			

Heterogeneity: Tau² = 0.22; Chi² = 8.39, df = 6 (P = 0.21); I² = 29%
Test for overall effect: Z = 0.78 (P = 0.43)

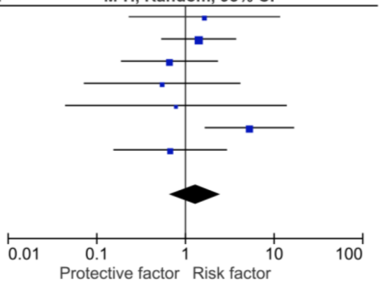

3.15 Figure S15. Forest plot showing the risk of ischemic complication associated with scalp tenderness.

Study or Subgroup	with ischemic event Events	with ischemic event Total	without ischemic event Events	without ischemic event Total	Weight	Odds Ratio M-H, Random, 95% CI
Chazal 2018	7	14	7	14	5.6%	1.00 [0.23, 4.40]
DeBoysson 2017	16	40	65	143	24.1%	0.80 [0.39, 1.63]
DeMornac 2021	8	39	50	213	17.4%	0.84 [0.36, 1.95]
Gonzalez-Gay 2009	2	8	99	279	4.7%	0.61 [0.12, 3.06]
Hayreh 1998	15	85	19	85	21.5%	0.74 [0.35, 1.59]
Parreau 2022	4	19	264	541	9.9%	0.28 [0.09, 0.85]
Penet 2023	2	14	97	271	5.3%	0.30 [0.07, 1.36]
Sun 2016	9	29	12	41	11.5%	1.09 [0.39, 3.06]
**Total (95% CI)**		**248**		**1587**	**100.0%**	**0.70 [0.50, 1.00]**
Total events	63		613			

Heterogeneity: Tau² = 0.00; Chi² = 5.14, df = 7 (P = 0.64); I² = 0%
Test for overall effect: Z = 1.97 (P = 0.05)

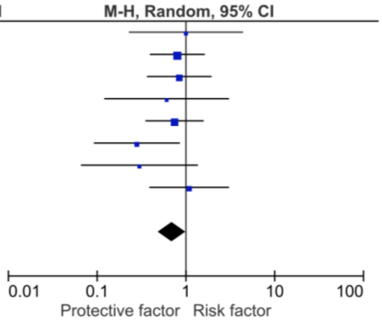

3.16 Figure S16. Forest plot showing the risk of ischemic complication associated with headache.

Study or Subgroup	with ischemic event Events	with ischemic event Total	without ischemic event Events	without ischemic event Total	Weight	Odds Ratio M-H, Random, 95% CI
Chazal 2018	10	14	10	14	3.6%	1.00 [0.19, 5.15]
Cid 1998	26	32	127	168	6.9%	1.40 [0.54, 3.64]
DeBoysson 2017	29	40	161	200	8.3%	0.64 [0.29, 1.39]
DeMornac 2021	19	39	131	214	9.1%	0.60 [0.30, 1.19]
Gonzalez-Gay 1998	59	69	139	170	8.3%	1.32 [0.61, 2.86]
Gonzalez-Gay 2009	5	8	238	279	4.2%	0.29 [0.07, 1.25]
Hayreh 1998	38	85	59	85	9.5%	0.36 [0.19, 0.67]
Hocevar 2020	48	61	157	234	9.2%	1.81 [0.93, 3.54]
Nesher 2004	29	43	103	132	8.4%	0.58 [0.27, 1.25]
Pariente 2019	15	18	74	111	4.9%	2.50 [0.68, 9.18]
Parreau 2022	10	19	446	541	7.1%	0.24 [0.09, 0.60]
Pego-Reigosa 2004	26	30	157	180	5.7%	0.95 [0.30, 2.98]
Penet 2023	7	14	181	271	6.1%	0.50 [0.17, 1.46]
Sun 2016	21	29	27	41	6.4%	1.36 [0.48, 3.85]
Zenone 2013	5	6	69	92	2.3%	1.67 [0.18, 15.02]
**Total (95% CI)**		**507**		**2732**	**100.0%**	**0.78 [0.54, 1.12]**
Total events	347		2079			

Heterogeneity: Tau² = 0.25; Chi² = 30.31, df = 14 (P = 0.007); I² = 54%
Test for overall effect: Z = 1.36 (P = 0.17)

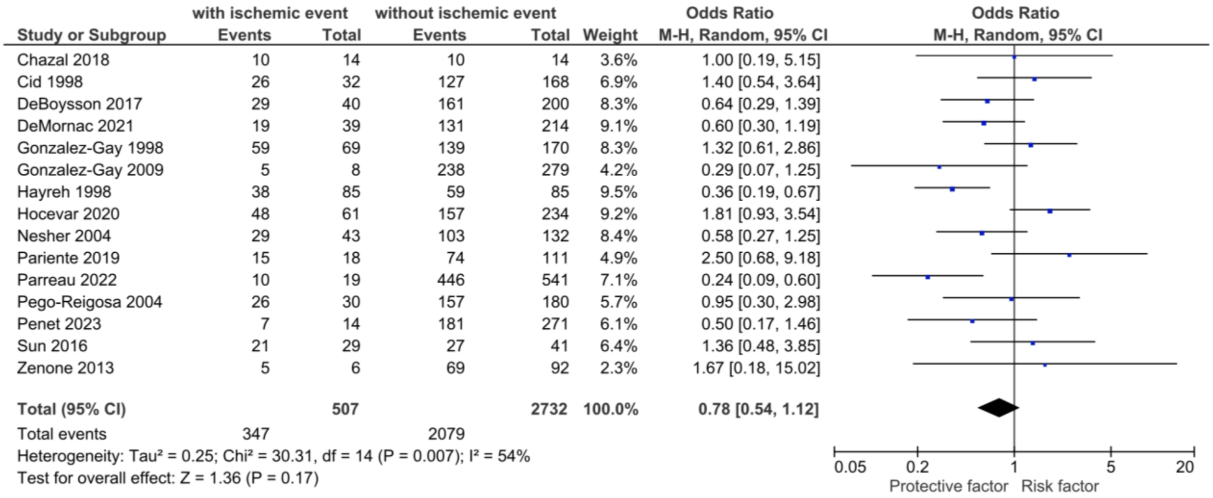

3.17 Figure S17. Forest plot showing the risk of ischemic complication associated with jaw claudication.

Study or Subgroup	with ischemic event Events	with ischemic event Total	without ischemic event Events	without ischemic event Total	Weight	Odds Ratio M-H, Random, 95% CI
Chazal 2018	5	14	7	14	3.5%	0.56 [0.12, 2.53]
Cid 1998	17	32	75	168	8.4%	1.41 [0.66, 3.00]
DeBoysson 2017	14	40	60	146	8.7%	0.77 [0.37, 1.60]
DeMornac 2021	8	39	60	214	7.6%	0.66 [0.29, 1.52]
Gonzalez-Gay 1998	32	66	58	166	10.4%	1.75 [0.98, 3.13]
Gonzalez-Gay 2009	2	8	113	279	3.1%	0.49 [0.10, 2.47]
Hayreh 1998	46	85	37	85	10.1%	1.53 [0.84, 2.80]
Hocevar 2020	40	61	84	234	10.2%	3.40 [1.88, 6.15]
Nesher 2004	12	43	19	132	7.7%	2.30 [1.01, 5.25]
Parreau 2022	4	19	181	541	5.4%	0.53 [0.17, 1.62]
Pego-Reigosa 2004	11	30	75	180	7.9%	0.81 [0.36, 1.80]
Penet 2023	6	14	98	271	5.6%	1.32 [0.45, 3.93]
Salvarani 2009	17	38	67	142	8.8%	0.91 [0.44, 1.86]
Zenone 2013	2	6	32	92	2.7%	0.94 [0.16, 5.40]
**Total (95% CI)**		**495**		**2664**	**100.0%**	**1.19 [0.87, 1.64]**
Total events	216		966			

Heterogeneity: Tau² = 0.17; Chi² = 25.36, df = 13 (P = 0.02); I² = 49%
Test for overall effect: Z = 1.09 (P = 0.27)

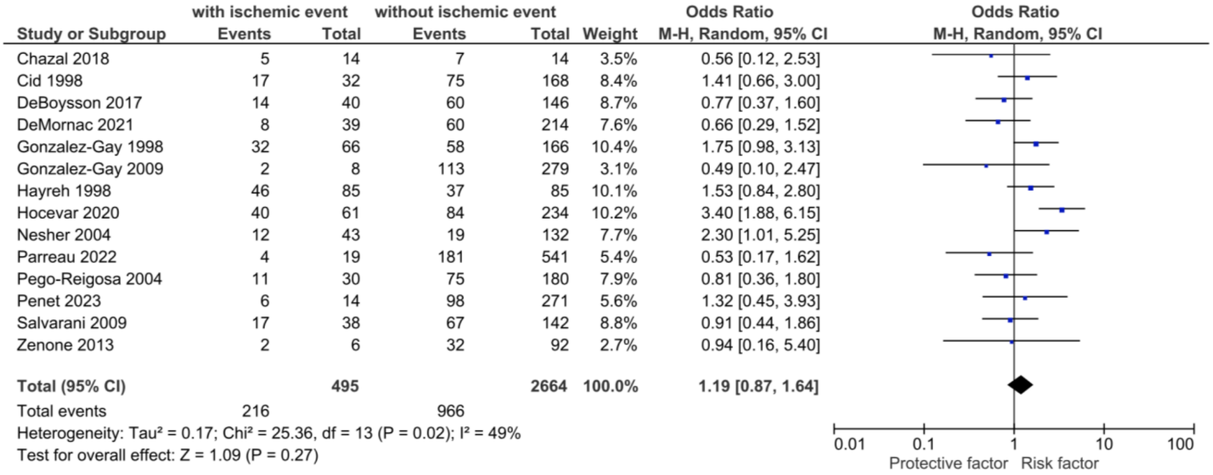

3.18 Figure S18. Forest plot showing the risk of ischemic complication associated with abnormal temporal artery on physical examination.

Study or Subgroup	with ischemic events: Events	with ischemic events: Total	without ischemic events: Events	without ischemic events: Total	Weight	Odds Ratio M-H, Random, 95% CI
Cid 1998	30	32	144	168	5.1%	2.50 [0.56, 11.15]
DeBoysson 2017	21	40	62	140	15.2%	1.39 [0.69, 2.81]
Gonzalez-Gay 1998	52	68	117	167	16.6%	1.39 [0.72, 2.66]
Gonzalez-Gay 2009	6	8	197	279	4.4%	1.25 [0.25, 6.32]
Hayreh 1998	14	85	22	85	14.1%	0.56 [0.27, 1.20]
Hocevar 2020	55	61	174	234	11.3%	3.16 [1.30, 7.71]
Parreau 2022	14	19	310	541	9.1%	2.09 [0.74, 5.87]
Pego-Reigosa 2004	22	30	137	180	11.5%	0.86 [0.36, 2.08]
Penet 2023	1	14	44	271	2.9%	0.40 [0.05, 3.11]
Sun 2016	8	29	9	41	8.4%	1.35 [0.45, 4.07]
Zenone 2013	0	6	25	92	1.5%	0.20 [0.01, 3.75]
**Total (95% CI)**		**392**		**2198**	**100.0%**	**1.27 [0.88, 1.82]**
Total events	223		1241			

Heterogeneity: Tau² = 0.10; Chi² = 13.80, df = 10 (P = 0.18); I² = 28%
Test for overall effect: Z = 1.27 (P = 0.20)

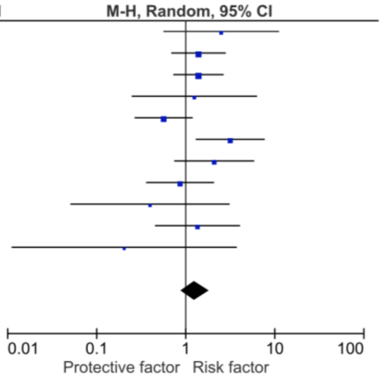

3.19 Figure S19. Forest plot showing the risk of ischemic complication associated with erythrocyte sedimentation rate (ESR; in mm by hours).

Study or Subgroup	with ischemic event: Mean [mm/H]	with ischemic event: SD [mm/H]	with ischemic event: Total	without ischemic event: Mean [mm/H]	without ischemic event: SD [mm/H]	without ischemic event: Total	Weight	Mean Difference IV, Random, 95% CI
Chazal 2018	76	33	14	79	41	14	1.8%	-3.00 [-30.57, 24.57]
Cid 1998	79.8	26.5	32	94.7	21	168	6.9%	-14.90 [-24.62, -5.18]
DeBoysson 2017	66.3	27.3	40	77.5	21.7	200	7.3%	-11.20 [-20.18, -2.22]
DeKeyser 1991	95.6	31	18	91.3	23	38	4.1%	4.30 [-11.78, 20.38]
DeMornac 2021	80.3	26	39	78.5	34	215	7.1%	1.80 [-7.54, 11.14]
Gonzalez-Gay 1998	93	26	69	95	25	170	8.5%	-2.00 [-9.19, 5.19]
Gonzalez-Gay 2009	82	20	8	94	23	279	4.8%	-12.00 [-26.12, 2.12]
Hayreh 1998	80	31	72	92	33	76	6.6%	-12.00 [-22.31, -1.69]
Hocevar 2020	76.3	28.1	61	86.7	38	234	7.6%	-10.40 [-18.97, -1.83]
Nesher 1998	98	27	18	102	27	83	4.9%	-4.00 [-17.76, 9.76]
Nesher 2004	105.9	22.2	43	102.1	28.4	132	7.8%	3.80 [-4.42, 12.02]
Parreau 2022	74.5	34.2	19	84	28.5	541	4.2%	-9.50 [-25.06, 6.06]
Pego-Reigosa 2004	92.1	23.2	30	93.2	22.3	180	7.4%	-1.10 [-10.02, 7.82]
Salvarani 2009	86	25	38	96	28	142	7.2%	-10.00 [-19.19, -0.81]
Sun 2016	81.6	21	29	101	25	41	6.3%	-19.40 [-30.22, -8.58]
Zenone 2013	85.9	9.3	6	78.6	22.2	92	7.5%	7.30 [-1.42, 16.02]
**Total (95% CI)**			**536**			**2605**	**100.0%**	**-5.70 [-9.71, -1.69]**

Heterogeneity: Tau² = 36.04; Chi² = 35.12, df = 15 (P = 0.002); I² = 57%
Test for overall effect: Z = 2.79 (P = 0.005)

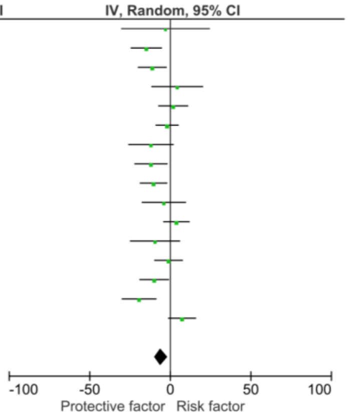

3.20 Figure S20. Forest plot showing the risk of ischemic complication associated with age at diagnosis.

Study or Subgroup	with ischemic events: Mean [années]	with ischemic events: SD [années]	with ischemic events: Total	without ischemic events: Mean [années]	without ischemic events: SD [années]	without ischemic events: Total	Weight	Mean Difference IV, Random, 95% CI
Chazal 2018	73.5	5	14	73.7	5.2	14	4.9%	-0.20 [-3.98, 3.58]
Cid 1998	75.5	5.5	32	73.8	5.8	168	8.1%	1.70 [-0.40, 3.80]
DeKeyser 1991	76	5	18	75	7	38	5.9%	1.00 [-2.21, 4.21]
DeMornac 2021	72.3	8.2	39	71.9	8.7	215	6.6%	0.40 [-2.42, 3.22]
Gonzalez-Gay 1998	73.6	6.8	69	73.4	6.6	170	8.5%	0.20 [-1.69, 2.09]
Gonzalez-Gay 2009	73.9	9.6	7	75.3	6.8	280	2.0%	-1.40 [-8.56, 5.76]
Hayreh 1998	76.2	7	85	73.8	7.2	85	8.0%	2.40 [0.27, 4.53]
Hocevar 2020	77.8	6	61	73	9.5	234	8.4%	4.80 [2.86, 6.74]
Nesher 1998	75.8	10	18	71.1	13	83	3.1%	4.70 [-0.70, 10.10]
Nesher 2004	75.3	9.5	43	73.6	8.1	132	6.0%	1.70 [-1.46, 4.86]
Pariente 2019	82.3	7.3	18	76.5	6.3	111	5.3%	5.80 [2.23, 9.37]
Parreau 2022	76.1	5.3	19	74.8	8.1	541	7.3%	1.30 [-1.18, 3.78]
Pego-Reigosa 2004	73.3	7.5	30	74.8	6.9	180	6.5%	-1.50 [-4.37, 1.37]
Penet 2023	70	9	14	72	9	257	3.6%	-2.00 [-6.84, 2.84]
Salvarani 2009	77	5	38	73	7	142	8.4%	4.00 [2.04, 5.96]
Sun 2016	63.8	9.2	29	66.1	6.9	41	4.7%	-2.30 [-6.26, 1.66]
Zenone 2013	74.6	7.2	6	75.4	5.5	92	2.8%	-0.80 [-6.67, 5.07]
**Total (95% CI)**			**540**			**2783**	**100.0%**	**1.48 [0.36, 2.60]**

Heterogeneity: Tau² = 2.92; Chi² = 38.57, df = 16 (P = 0.001); I² = 59%
Test for overall effect: Z = 2.58 (P = 0.010)

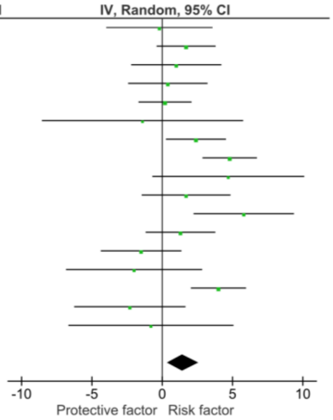

3.21 Figure S21. Forest plot showing the risk of ischemic complication associated with C-reactive protein (CRP) levels (mg/L).

Study or Subgroup	with ischemic event Mean [mg/L]	SD [mg/L]	Total	without ischemic event Mean [mg/L]	SD [mg/L]	Total	Weight	Mean Difference IV, Random, 95% CI
Chazal 2018	49.5	50.1	14	122	121	14	3.1%	-72.50 [-141.10, -3.90]
DeBoysson 2017	83.8	39.3	40	151	65.7	200	12.5%	-67.20 [-82.41, -51.99]
DeMornac 2021	112	74	39	105	79	215	9.7%	7.00 [-18.51, 32.51]
Hayreh 1998	82	140	14	74	76	31	2.5%	8.00 [-70.06, 86.06]
Hocevar 2020	64	52.4	61	89.3	75.3	234	12.2%	-25.30 [-41.61, -8.99]
Pariente 2019	88.3	50.3	18	121.5	56.3	111	9.7%	-33.20 [-58.69, -7.71]
Parreau 2022	68	57	19	94	66	541	9.5%	-26.00 [-52.23, 0.23]
Penet 2023	54	22	14	74	13	257	13.3%	-20.00 [-31.63, -8.37]
Salvarani 2009	72	45	38	104	63	142	11.8%	-32.00 [-49.67, -14.33]
Sun 2016	57.6	49.9	29	84	57	41	9.8%	-26.40 [-51.58, -1.22]
Zenone 2013	107.5	52.5	6	132.3	59.8	92	5.8%	-24.80 [-68.55, 18.95]
**Total (95% CI)**			**292**			**1878**	**100.0%**	**-29.01 [-42.61, -15.41]**

Heterogeneity: Tau² = 325.92; Chi² = 36.92, df = 10 (P < 0.0001); I² = 73%
Test for overall effect: Z = 4.18 (P < 0.0001)

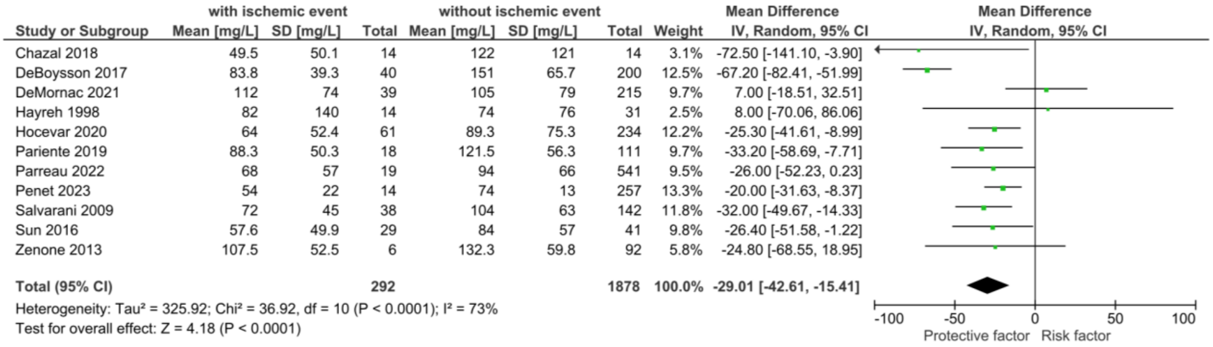

3.22 Figure S22. Forest plot showing the risk of ischemic complication associated with hemoglobin level (g/dL).

Study or Subgroup	with ischemic events Mean [g/dL]	SD [g/dL]	Total	without ischemic events Mean [g/dL]	SD [g/dL]	Total	Weight	Mean Difference IV, Random, 95% CI
Chazal 2018	11.9	1.1	14	11.4	1.9	14	4.5%	0.50 [-0.65, 1.65]
Cid 1998	12.6	1.5	32	11	1.3	168	9.0%	1.60 [1.04, 2.16]
DeBoysson 2017	12.4	1.7	40	11.5	1.3	200	9.0%	0.90 [0.34, 1.46]
DeKeyser 1991	10.9	2.2	18	11.4	1.8	38	4.4%	-0.50 [-1.67, 0.67]
DeMornac 2021	11	1.7	39	11.2	1.6	215	8.8%	-0.20 [-0.77, 0.37]
Gonzalez-Gay 2009	13.2	1.5	8	11.7	1.6	279	5.0%	1.50 [0.44, 2.56]
Hocevar 2020	12	1.6	61	11.6	1.7	234	10.0%	0.40 [-0.06, 0.86]
Nesher 1998	11.5	1.4	18	11.2	1.8	83	7.1%	0.30 [-0.45, 1.05]
Nesher 2004	11.4	1.4	43	11.2	1.5	132	9.7%	0.20 [-0.29, 0.69]
Parreau 2022	12	1.8	19	11.6	1.7	541	6.6%	0.40 [-0.42, 1.22]
Pego-Reigosa 2004	11.8	1.6	30	11.7	1.6	180	8.4%	0.10 [-0.52, 0.72]
Penet 2023	12.1	0.8	14	11.7	0.4	257	10.4%	0.40 [-0.02, 0.82]
Sun 2016	11.5	1.4	29	10.8	1.8	41	7.2%	0.70 [-0.05, 1.45]
**Total (95% CI)**			**365**			**2382**	**100.0%**	**0.49 [0.19, 0.79]**

Heterogeneity: Tau² = 0.18; Chi² = 33.01, df = 12 (P = 0.0010); I² = 64%
Test for overall effect: Z = 3.21 (P = 0.001)

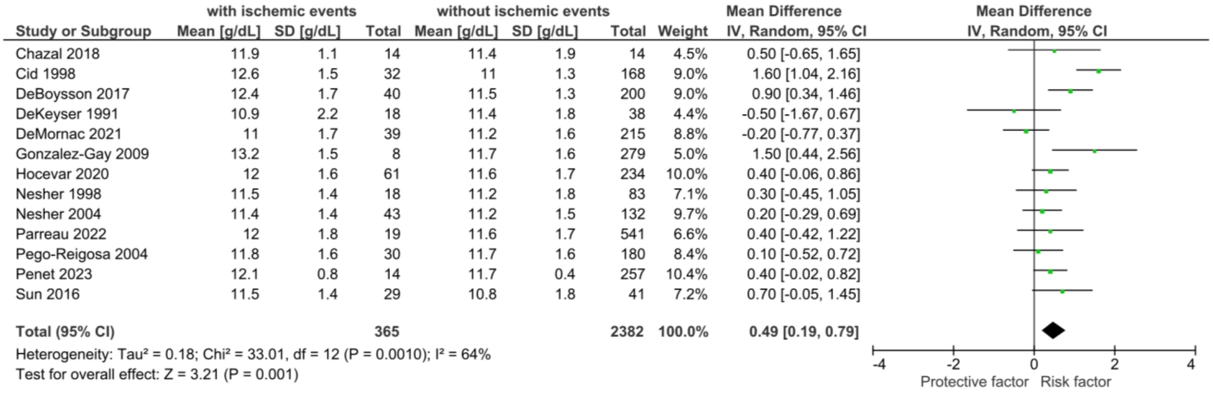

3.23 Figure S23. Forest plot showing the risk of ischemic complication associated with diagnosis delay (weeks).

Study or Subgroup	with ischemic events Mean [weeks]	SD [weeks]	Total	without ischemic events Mean [weeks]	SD [weeks]	Total	Weight	Mean Difference IV, Random, 95% CI
Gonzalez-Gay 2009	12.6	17.6	7	9.8	10.3	280	3.7%	2.80 [-10.29, 15.89]
Hocevar 2020	34.7	34.9	61	40.3	36.6	234	6.4%	-5.60 [-15.53, 4.33]
Nesher 1998	5.5	6.5	18	7.6	6	83	58.9%	-2.10 [-5.37, 1.17]
Parreau 2022	81	63	19	80	88	541	0.7%	1.00 [-28.28, 30.28]
Pego-Reigosa 2004	11.1	12.2	30	10.3	11.4	180	28.8%	0.80 [-3.87, 5.47]
Sun 2016	17.5	44.9	29	12.3	37.6	41	1.6%	5.20 [-14.79, 25.19]
**Total (95% CI)**			**164**			**1359**	**100.0%**	**-1.17 [-3.68, 1.34]**

Heterogeneity: Tau² = 0.00; Chi² = 2.52, df = 5 (P = 0.77); I² = 0%
Test for overall effect: Z = 0.91 (P = 0.36)

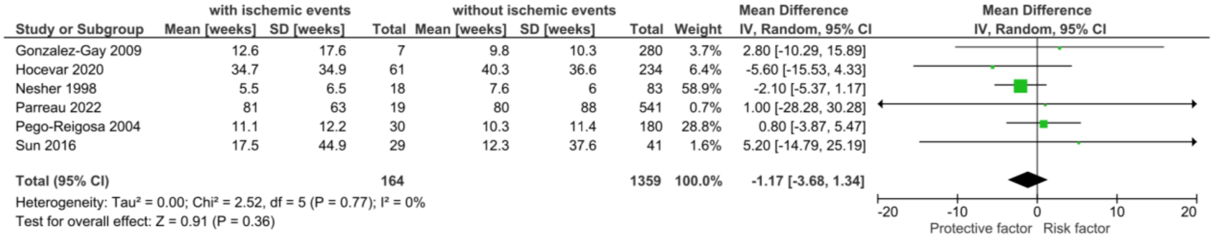

3.24 Figure S24 Forest plot showing the risk of ischemic complication associated with platelet counts (G/L).

	with ischemic event			without ischemic event				Mean Difference
Study or Subgroup	Mean [giga/L]	SD [giga/L]	Total	Mean [giga/L]	SD [giga/L]	Total	Weight	IV, Random, 95% CI
Chazal 2018	453	159	14	414	150	14	7.8%	39.00 [-75.50, 153.50]
Cid 1998	323.5	130.5	32	424.5	103.7	168	13.0%	-101.00 [-148.86, -53.14]
DeBoysson 2017	455.5	214.5	40	457.8	131.2	200	11.3%	-2.30 [-71.22, 66.62]
DeKeyser 1991	475	125	18	338	96	38	11.6%	137.00 [71.68, 202.32]
DeMornac 2021	414	169	39	412	134	215	12.4%	2.00 [-53.98, 57.98]
Gonzalez-Gay 2009	350	139	8	404	131	279	9.0%	-54.00 [-151.54, 43.54]
Nesher 2004	445	94	43	452	173	132	13.6%	-7.00 [-47.75, 33.75]
Parreau 2022	453	196	19	430	154	541	9.6%	23.00 [-66.08, 112.08]
Sun 2016	360	122	29	366	151	41	11.7%	-6.00 [-70.09, 58.09]
**Total (95% CI)**			**242**			**1628**	**100.0%**	**1.48 [-44.33, 47.28]**

Heterogeneity: Tau² = 3597.23; Chi² = 35.56, df = 8 (P < 0.0001); I² = 78%
Test for overall effect: Z = 0.06 (P = 0.95)

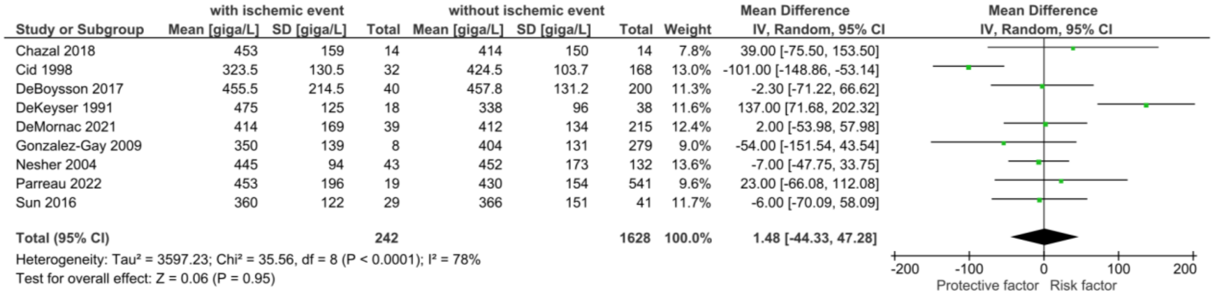

3.25 Figure S24 Forest plot showing the risk of ischemic complication associated with albumin (g/L).

	with ischemic event			without ischemic event				Mean Difference
Study or Subgroup	Mean [g/L]	SD [g/L]	Total	Mean [g/L]	SD [g/L]	Total	Weight	IV, Random, 95% CI
Cid 1998	3.9	1.1	32	3.4	0.7	168	50.9%	0.50 [0.10, 0.90]
Gonzalez-Gay 2009	3.16	0.61	8	3.3	0.6	279	49.1%	-0.14 [-0.57, 0.29]
**Total (95% CI)**			**40**			**447**	**100.0%**	**0.19 [-0.44, 0.81]**

Heterogeneity: Tau² = 0.16; Chi² = 4.63, df = 1 (P = 0.03); I² = 78%
Test for overall effect: Z = 0.58 (P = 0.56)

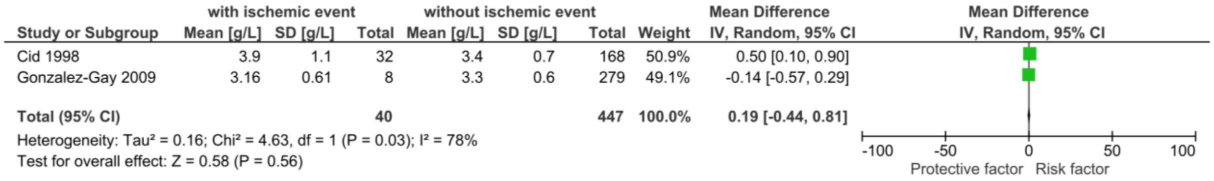

3.26 Figure S26 Forest plot showing the risk of ischemic complication associated with atrial fibrillation.

	with ischemic event		without ischemic event			Odds Ratio
Study or Subgroup	Events	Total	Events	Total	Weight	M-H, Random, 95% CI
Gonzalez-Gay 2009	1	8	21	279	8.3%	1.76 [0.21, 14.95]
Hocevar 2020	15	61	26	234	75.6%	2.61 [1.28, 5.31]
Penet 2023	2	14	33	271	16.1%	1.20 [0.26, 5.61]
**Total (95% CI)**		**83**		**784**	**100.0%**	**2.23 [1.20, 4.13]**
Total events	18		80			

Heterogeneity: Tau² = 0.00; Chi² = 0.85, df = 2 (P = 0.65); I² = 0%
Test for overall effect: Z = 2.54 (P = 0.01)

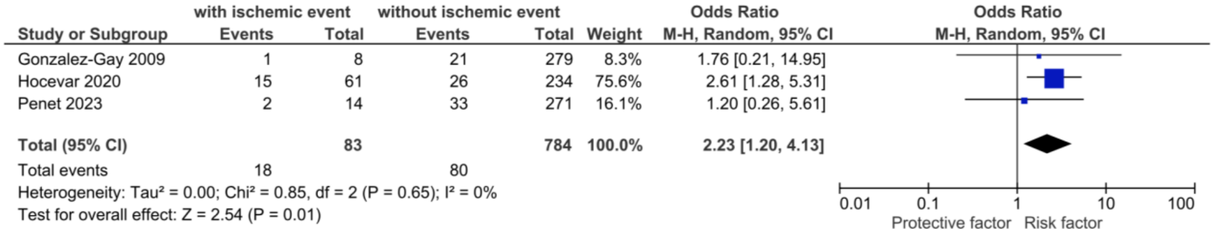

4 Availability

All data generated by this work are available on request.

5 References

Berger, C.T., Wolbers, M., Meyer, P., Daikeler, T., and Hess, C. (2009). High incidence of severe ischaemic complications in patients with giant cell arteritis irrespective of platelet count and size, and platelet inhibition. *Rheumatology (Oxford)* 48**,** 258-261.

Chazal, T., Couture, P., Rosso, C., Haroche, J., Leger, A., Hervier, B., Deltour, S., Rufat, P., Amoura, Z., and Cohen Aubart, F. (2018). Cerebrovascular events are associated with lower survival in giant cell arteritis: A case-controlled multicenter study. *Joint Bone Spine* 85**,** 383-385.

Cid, M.C., Font, C., Oristrell, J., De La Sierra, A., Coll-Vinent, B., Lopez-Soto, A., Vilaseca, J., Urbano-Marquez, A., and Grau, J.M. (1998). Association between strong inflammatory response and low risk of developing visual loss and other cranial ischemic complications in giant cell (temporal) arteritis. *Arthritis Rheum* 41**,** 26-32.

De Boysson, H., Liozon, E., Lariviere, D., Samson, M., Parienti, J.J., Boutemy, J., Maigne, G., Martin Silva, N., Ly, K., Touze, E., Bonnotte, B., Aouba, A., Sacre, K., and Bienvenu, B. (2017). Giant Cell Arteritis-related Stroke: A Retrospective Multicenter Case-control Study. *J Rheumatol* 44**,** 297-303.

De Keyser, J., De Klippel, N., and Ebinger, G. (1991). Thrombocytosis and ischaemic complications in giant cell arteritis. *BMJ* 303**,** 825.

De Mornac, D., Espitia, O., Neel, A., Connault, J., Masseau, A., Espitia-Thibault, A., Artifoni, M., Achille, A., Wahbi, A., Lacou, M., Durant, C., Pottier, P., Perrin, F., Graveleau, J., Hamidou, M., Hardouin, J.B., and Agard, C. (2021). Large-vessel involvement is predictive of multiple relapses in giant cell arteritis. *Ther Adv Musculoskelet Dis* 13**,** 1759720X211009029.

Gonzalez-Gay, M.A., Blanco, R., Rodriguez-Valverde, V., Martinez-Taboada, V.M., Delgado-Rodriguez, M., Figueroa, M., and Uriarte, E. (1998). Permanent visual loss and cerebrovascular accidents in giant cell arteritis: predictors and response to treatment. *Arthritis Rheum* 41**,** 1497-1504.

Gonzalez-Gay, M.A., Vazquez-Rodriguez, T.R., Gomez-Acebo, I., Pego-Reigosa, R., Lopez-Diaz, M.J., Vazquez-Trinanes, M.C., Miranda-Filloy, J.A., Blanco, R., Dierssen, T., Gonzalez-Juanatey, C., and Llorca, J. (2009). Strokes at time of disease diagnosis in a series of 287 patients with biopsy-proven giant cell arteritis. *Medicine (Baltimore)* 88**,** 227-235.

Hayreh, S.S., Podhajsky, P.A., and Zimmerman, B. (1998). Ocular manifestations of giant cell arteritis. *Am J Ophthalmol* 125**,** 509-520.

Hocevar, A., Jese, R., Tomsic, M., and Rotar, Z. (2020). Risk factors for severe cranial ischaemic complications in giant cell arteritis. *Rheumatology (Oxford)* 59**,** 2953-2959.

Lee, M.S., Smith, S.D., Galor, A., and Hoffman, G.S. (2006). Antiplatelet and anticoagulant therapy in patients with giant cell arteritis. *Arthritis Rheum* 54**,** 3306-3309.

Liozon, E., Herrmann, F.R., Ly, K., Jauberteau, M.O., Loustaud, V., Soria, P., Robert, P.Y., Liozon, F., and Vidal, E. (2001). [Risk factors for irreversible cerebral ischemia complications from Horton's disease: prospective study of 178 patients]. *Rev Med Interne* 22**,** 30-41.

Nesher, G., Berkun, Y., Mates, M., Baras, M., Nesher, R., Rubinow, A., and Sonnenblick, M. (2004). Risk factors for cranial ischemic complications in giant cell arteritis. *Medicine (Baltimore)* 83**,** 114-122.

Nesher, G., and Sonnenblick, M. (1998). No association between the inflammatory response and the risk of developing irreversible cranial ischemic complications: comment on the article by Cid et al. *Arthritis Rheum* 41**,** 2088-2089.

Pariente, A., Guedon, A., Alamowitch, S., Thietart, S., Carrat, F., Delorme, S., Capron, J., Cacciatore, C., Soussan, M., Dellal, A., Fain, O., and Mekinian, A. (2019). Ischemic stroke in giant-cell arteritis: French retrospective study. *J Autoimmun* 99**,** 48-51.
Parreau, S., Dumonteil, S., Montoro, F.M., Gondran, G., Bezanahary, H., Palat, S., Ly, K.H., Fauchais, A.L., and Liozon, E. (2022). Giant cell arteritis-related stroke in a large inception cohort: A comparative study. *Semin Arthritis Rheum* 55**,** 152020.
Pego-Reigosa, R., Garcia-Porrua, C., Pineiro, A., Dierssen, T., Llorca, J., and Gonzalez-Gay, M.A. (2004). Predictors of cerebrovascular accidents in giant cell arteritis in a defined population. *Clin Exp Rheumatol* 22**,** S13-17.
Penet, T., Lambert, M., Baillet, C., Outteryck, O., Henon, H., Morell-Dubois, S., Hachulla, E., Launay, D., and Pokeerbux, M.R. (2023). Giant cell arteritis-related cerebrovascular ischemic events: a French retrospective study of 271 patients, systematic review of the literature and meta-analysis. *Arthritis Res Ther* 25**,** 116.
Salvarani, C., Della Bella, C., Cimino, L., Macchioni, P., Formisano, D., Bajocchi, G., Pipitone, N., Catanoso, M.G., Restuccia, G., Ghinoi, A., and Boiardi, L. (2009). Risk factors for severe cranial ischaemic events in an Italian population-based cohort of patients with giant cell arteritis. *Rheumatology (Oxford)* 48**,** 250-253.
Sun, F., Ma, S., Zheng, W., Tian, X., and Zeng, X. (2016). A Retrospective Study of Chinese Patients With Giant Cell Arteritis (GCA): Clinical Features and Factors Associated With Severe Ischemic Manifestations. *Medicine (Baltimore)* 95**,** e3213.
Wan, X., Wang, W., Liu, J., and Tong, T. (2014). Estimating the sample mean and standard deviation from the sample size, median, range and/or interquartile range. *BMC Med Res Methodol* 14**,** 135.
Zenone, T., and Puget, M. (2013). Characteristics of cerebrovascular accidents at time of diagnosis in a series of 98 patients with giant cell arteritis. *Rheumatol Int* 33**,** 3017-3023.
